# Lignin bioconversion based on genome mining for ligninolytic genes in *Erwinia billingiae* QL-Z3

**DOI:** 10.1186/s13068-024-02470-z

**Published:** 2024-02-15

**Authors:** Shuting Zhao, Dongtao Deng, Tianzheng Wan, Jie Feng, Lei Deng, Qianyi Tian, Jiayu Wang, Umm E. Aiman, Balym Mukhaddi, Xiaofeng Hu, Shaolin Chen, Ling Qiu, Lili Huang, Yahong Wei

**Affiliations:** 1 State Key Laboratory of Crop Stress Biology for Arid Areas, College of Life Sciences, Biomass Energy Center for Arid and Semi-Arid Lands, Northwest A&F University, Yangling, Shaanxi, 712100 People’s Republic of China; 2 Vrije University Amsterdam, De Boelelaan 1105, 1081 HV Amsterdam, Netherlands; 3 Shanghai Personal Biotechnology Co., Ltd, Shanghai, 20030 People’s Republic of China; 4 College of Mechanical and Electronic Engineering, The West Scientific Observing and Experimental Station of Rural Renewable Energy Exploitation and Utilization of the Ministry of Agriculture, Northwest A&F University, Yangling, Shaanxi, 712100 People’s Republic of China; 5 State Key Laboratory of Crop Stress Biology for Arid Areas, College of Plant Protection, Northwest A&F University, Yangling, Shaanxi, 712100 People’s Republic of China

**Keywords:** Biomass utilization, Ligninolytic gene, Genetic modification, Lignin degradation product, Degradation mechanism

## Abstract

**Background:**

Bioconversion of plant biomass into biofuels and bio-products produces large amounts of lignin. The aromatic biopolymers need to be degraded before being converted into value-added bio-products. Microbes can be environment-friendly and efficiently degrade lignin. Compared to fungi, bacteria have some advantages in lignin degradation, including broad tolerance to pH, temperature, and oxygen and the toolkit for genetic manipulation.

**Results:**

Our previous study isolated a novel ligninolytic bacterial strain *Erwinia billingiae* QL-Z3. Under optimized conditions, its rate of lignin degradation was 25.24% at 1.5 g/L lignin as the sole carbon source. Whole genome sequencing revealed 4556 genes in the genome of QL-Z3. Among 4428 protein-coding genes are 139 CAZyme genes, including 54 glycoside hydrolase (GH) and 16 auxiliary activity (AA) genes. In addition, 74 genes encoding extracellular enzymes are potentially involved in lignin degradation. Real-time PCR quantification demonstrated that the expression of potential ligninolytic genes were significantly induced by lignin. 8 knock-out mutants and complementary strains were constructed. Disruption of the gene for *ELAC_205* (laccase) as well as *EDYP_48* (Dyp-type peroxidase), *ESOD_1236* (superoxide dismutase), *EDIO_858* (dioxygenase), *EMON_3330* (monooxygenase), or *EMCAT_3587* (manganese catalase) significantly reduced the lignin-degrading activity of QL-Z3 by 47–69%. Heterologously expressed and purified enzymes further confirmed their role in lignin degradation. Fourier transform infrared spectroscopy (FTIR) results indicated that the lignin structure was damaged, the benzene ring structure and groups of macromolecules were opened, and the chemical bond was broken under the action of six enzymes encoded by genes. The abundant enzymatic metabolic products by EDYP_48, ELAC_205 and ESOD_1236 were systematically analyzed via liquid chromatography–mass spectrometry (LC–MS) analysis, and then provide a speculative pathway for lignin biodegradation. Finally, The activities of ligninolytic enzymes from fermentation supernatant, namely, LiP, MnP and Lac were 367.50 U/L, 839.50 U/L, and 219.00 U/L by orthogonal optimization.

**Conclusions:**

Our findings provide that QL-Z3 and its enzymes have the potential for industrial application and hold great promise for the bioconversion of lignin into bioproducts in lignin valorization.

**Supplementary Information:**

The online version contains supplementary material available at 10.1186/s13068-024-02470-z.

## Introduction

Lignin is the second most abundant renewable carbon source on earth, next to cellulose. It is a crosslinked phenolic polymer mainly comprised of three constituent monomers, 4-hydroxyphenyl (H), guaiacyl (4-hydroxy-3-methoxyphenyl, G), and syringyl (4-hydroxy-3,5-dimethoxyphenyl, S). The complex of these components is cross-linked together through carbon–carbon, ester, and ether linkages [1]. Recent genetic evidence indicates that cross-linking between lignin and polysaccharides has a substantial impact on plant cell wall recalcitrance [2]. Lignin inhibits plant biomass digestion in several ways, mainly by preventing microbes and enzymes from gaining access to cellulose, but also by binding digestive enzymes or releasing inhibitory breakdown products [3]. In agricultural production, a large amount of lignin exists in the waste straw, which is difficult to degrade and damage the soil environment [4]; lignin is also a by-product of wood hydrolysis and paper industry application [5]. Statistically, pulp and paper facilities produce 50–70 million tons of lignin per year, more than 695.7 million cubic meters in the global papermaking wastewater discharge, of which lignin accounts for 600–1000 mg/L in the black liquor of papermaking, accounting for 47.4% and 59.4% of chemical oxygen demand and chroma of papermaking wastewater [6, 7]. Compared with chemical and physical methods, biological process for the degradation of lignin is regarded as eco-friendly, cost-effective and sustainable [6]. Therefore, finding efficient lignin-degrading strains is increasingly being considered an effective method for the biological pretreatment of lignocellulosic biomass.

As an alternative raw material, lignin has enormous potential to replace diminishing fossil-based resources for the sustainable production of many chemicals and materials, including valuable aromatic and non-aromatic chemicals, such as vanillin [8], aromatic carboxylic acids including cis-muconic acid, adipic acid, polyols, lipids, and polyhydroxyalkanoates (PHAs). In the past few decades, researches on the microbial degradation of lignin mainly focused on the degradation of lignin by fungi [9]. Compared with fungi, bacteria can adapt to extreme environments such as strong acid, strong alkali, and abundant temperatures because they have better environmental adaptability and abundant biodiversity. In addition, bacteria provide a vast and diverse toolbox for mining and selecting ligninolytic bacterial strains and enzymes and for the metabolic engineering of bacterial cell factories for effective biomass conversion processes. Therefore, bacteria are currently in the spotlight as promising candidates for novel biomass conversion strategies due to their wide functional diversity and versatility [10].

Recently several bacterial strains, such as *Streptomyces* [11], *Burkholderia* [12], *Bacillus* [13], *Pseudomonas* [14], and *Microbacterium* [15], have demonstrated lignin degradation abilities. Studies on *Sphingomonas paucimobilis* SYK-6 have shown that this strain has a wide assimilation ability of lignin-derived biaryl groups [16]. Bacterial ligninolytic enzymes can be divided into two categories: lignin-modifying enzymes (LME) and lignin depolymerizing auxiliary enzymes (LDA). LME includes lignin peroxidase (LiP, EC 1.11.1.14), manganese peroxidase (MnP, EC 1.11.1.13), versatile peroxidase (EC 1.11.1.16), laccase (Lac, EC 1.10.3.2), and Dyp-decolorizing peroxidase (EC 1.11.1.19) [17]. All members of this group belong to class II of the catalase superfamily and participated in the depolymerization of large lignin polymers to produce phenoxy intermediates [18, 19]. LDA enzyme includes several enzymes, such as cellobiose dehydrogenase (EC 1.1.99.18), glyoxal oxidase (EC 1.2.3.5), superoxide dismutase (EC.1.15.1.1) and glucose dehydrogenase (EC 1.1.99.10), which are incapable of degrading lignin on their own but are required for ligninolytic enzymes to degrade lignin. Moreover, other enzymes, such as oxygenase, that have not been classified, was also found to play a role in lignin degradation [20].

Here, we report the complete genome sequence of a novel lignin degrader, *Erwinia billingiae* QL-Z3. Based on the whole-genome annotation, eight knock-out mutants and complementary strains were constructed to confirm the function of lignin degradation genes. The heterogonous expression of six selected genes derived from QL-Z3 was performed to clarify its enzymatic properties. Then the structure and composition of the products of oxidative degrading enzymes were detected by FTIR and LC–MS. Enzymatic hydrolysis products on lignin degradation and its mechanism were analyzed to provide a speculative pathway for lignin biodegradation. Finally, the enzyme activities (LiP, MnP, Lac) from the fermentation medium of stain QL-Z3 were optimized. Taken together, the results suggest that diverse oxidative enzymes are involved in lignin degradation by QL-Z3, making this strain an ideal source of ligninolytic and lignocellulolytic enzymes and a good candidate for plant cell wall/biomass hydrolysis. Strain QL-Z3 has a significant potential for biological valorization of lignin bioconversion.

## Methods

### Experimental strain and SEM morphology

*E. billingiae* QL-Z3 was previously isolated from soil samples taken from the Qinling Mountains in China and stored in China General Microbiological Culture Collection Center (CGMCC 1.16623) [21]. Strain QL-Z3 was cultured in liquid Luria–Bertani (LB) medium at 30 °C for 12 h and the cells were immobilized with 2.5% (V/V) glutaraldehyde for 2 h, then washed with 0.1 M phosphate buffered saline (PBS, pH7.3) for three times. Cells were dehydrated and fixed with ethanol concentration gradients (V/V) of 50%, 70%, 80%, 90%, and 100% to prepared into tablets, then observed morphological features of the strain by SEM.

### Optimization of degradation rate

The bacteria QL-Z3 were cultured in LB medium at 30 °C for 72 h, and then the cells were collected by centrifugation. After resuspension in 100 µL phosphate buffered saline (PBS, pH 6.8), cells were transferred and cultured in different pH (5, 7, 9, 11), lignin concentration (0.5 g/L, 1.0 g/L, 1.5 g/L, 2.0 g/L) and nitrogen source [tryptone, (NH_4_)_2_SO_4_, NH_4_NO_3_, NaNO_3_] gradients lignin medium for 72 h (no bacteria inoculation treatments as blank control). For the analysis of lignin degradation, *E. billingiae* QL-Z3 in lignin medium was collected by centrifugation, and residual lignin in the medium was monitored by UV–visible spectrophotometry at 280 nm (OD_280_) as described by Li et al. [22]. The influence of pH, lignin concentration and nitrogen source on the degradation rate of lignin were estimated through variations of single factors and orthogonal optimization.

### Genome of *Erwinia billingiae* QL-Z3

#### Whole-genome sequencing and assembly

The total DNA of strain QL-Z3 was extracted by the CTAB (Cetyltrimethylammonium Bromide) method. Whole-genome sequencing was performed using single-molecule real-time (SMRT) and Illumina-based sequencing data assembly technology in Shanghai Personalbio Technology Co., Ltd. Using HGAP4 software to assemble the off-machine data and obtain contig sequence. After obtaining the second generation, pilon software was used to correct the contig results of the third generation (version 1.22), and the complete sequence was spliced. Finally, the assembled sequences were evaluated.

GeneMarkS gene prediction software (version 4.32) was used for gene prediction of QL-Z3 whole gene sequence. Based on the frequency table matrix used by nucleic acid in the sequence, the software predicted the potential coding regions of the sequence, improved the identification of gene translation sites, and reduced the false positive rate.

#### Gene annotation and prediction of ligninolytic and CAZyme genes

Trnascan-se 3.1 software and Barrnap (0.9-Dev) software were used to predict tRNA genes and rRNA genes in QL-Z3. The antibiotic resistance genes of the strain were obtained by comparison analysis in the CARD (Comprehensive Antibiotic Research Database), and the software HMMSCAN (3.1B2, February 2015) was used to predict the Existence of CAZy enzyme encoded by genes in the genome sequence [23]. The circle maps of nucleosomes and plasmids were drawn through Photoshop software. Sequence alignment of protein-coding genes was compared with the NR database to annotate gene function, select critical value 1e-6 for function discrimination. The results of functional annotation, sequence BLAST searches, and related reports were integrated to predict lignin-degrading genes in QL-Z3. Protein domains were searched against the InterPro database (http://www.ebi.ac.uk/interpro/).

### Quantification of mRNA levels

*Erwinia* *billingiae* QL-Z3 were inoculated into 1.5 g/L alkaline lignin medium, without carbon source medium and glucose medium and incubated under 180 rpm, at 30 °C. Total RNA was extracted according to Trizol (Takara) reagent instructions at 6 h and 72 h, then using PrimeScript ™ RT reagent Kit with gDNA Eraser to synthesize cDNA. RT-PCR primers were designed according to genes and reference genes, respectively. Using StepOne Plus2.2.2 software to collect the Ct values of cycle number, the ΔCt method was analyzed and calculated the relative expression levels of corresponding target genes in different samples. The calculation formula is ${{{\text{Ratio}} = \left( {{\text{E}}_{{{\text{target}}}} } \right)*\Delta {\text{CP}}_{{{\text{target}}\,\,\left( {{\text{control}} - {\text{treatment}}} \right)}} } \mathord{\left/ {\vphantom {{{\text{Ratio}} = \left( {{\text{E}}_{{{\text{target}}}} } \right)*\Delta {\text{CP}}_{{{\text{target}}\,\,\left( {{\text{control}} - {\text{treatment}}} \right)}} } {\left( {{\text{E}}_{{{\text{racA}}}} } \right)*\Delta {\text{CP}}_{{{\text{recA}}\,\left( {{\text{control}} - {\text{treatment}}} \right)}} }}} \right. \kern-0pt} {\left( {{\text{E}}_{{{\text{racA}}}} } \right)*\Delta {\text{CP}}_{{{\text{recA}}\,\left( {{\text{control}} - {\text{treatment}}} \right)}} }}$.

### Functional verification of lignin degrading genes

#### Construction of knock-out mutants and complementary strains

To further characterize the ligninolytic genes, eight genes were deleted using homologous recombination. The amplicon containing deletion was digested ligated into the vector pDM4 at sites XbaI and XhoI, resulting in the recombinant plasmid designated. The primers used in this study are shown in Additional file 1: Table S4. Then first transformed into *E. coli* S17-1 and subsequently into QL-Z3 via conjunction with *E. coli* S17-1. Integrants in QL-Z3 were selected with chloramphenicol (50 μg/mL). Counterselection with sucrose was performed to get positive clones and then verified by sequencing of PCR amplicons.

Eight deletion genes were amplified from strain QL-Z3. The PCR product was digested with EcoRI/XbaI and inserted into the EcoRI/XbaI sites of pKT100 (Additional file 1: Table S5). The recombinant was obtained using electric shock transformation.

#### Determination of degradation rate, enzyme activity and structural changes

To measure the degradation rate, the supernatant sample was collected by centrifugation of the reaction mixture at 8000 rpm for 5 min, then divided in two, one was used to determine the degradation rate and enzyme activity. Alkali lignin degradation-rate = (C1−C2)/C1*100%. (In the formula, C1 is the concentration of alkali lignin at time 0, and C2 is the concentration of alkali lignin after treatment with strain QL-Z3 for 3 days, alkali lignin concentration was calculated by standard curve equation: *y* = 1.1765 x + 0.035, *R*^2^ = 0.998). Enzyme activities of lignin peroxidase, manganese peroxidase [24], laccase [25], dioxygenase [26], superoxide dismutase [27] and manganese catalase [28] were measured as described. Another one was dried to a fixed weight, and the dried sample was analyzed by FT-IR spectroscopy.

### Heterologous expression and characterization

The expressed gene was amplified from QL-Z3 using the primer pair in Additional file 1: Table S2. The PCR product was digested with XhoI/XbaI and inserted into the XhoI/XbaI sites of plasmid pColdI. The transformant was selected against ampicillin (50 μg/mL) and then verified by sequencing of PCR amplicons. Recombinant pColdI plasmids were transformed into competent *E. coli* BL21(DE3) cells and purified using an Ni-Bestarose fast flow column (GE Healthcare, USA).

After measuring the enzyme activity encoded by genes *EDYP_48* and *ELAC_205*, the effects of temperature and pH with ABTS as the substrate were investigated. The maximum enzyme activity was taken as 100%, and the relative activities were calculated. The Enzyme kinetic parameters Km, Kcat, and Vmax value were determined by measuring the oxidation of ABTS.

### Enzymatic hydrolysis rate and products analysis of alkali lignin

Alkali lignin (1 g/L) and 0.2 mg enzyme solution were added into 3 mL sodium acetate buffer at pH 7.0 and then incubated in a 37 °C shaking bath. After 24 h, the enzymatic hydrolysis reduction of the alkali lignin solution was measured at 280 nm.

The LC analysis was performed on a Vanquish UHPLC System (Thermo Fisher Scientific, USA). Chromatography was carried out with an ACQUITY UPLC ^®^ HSS T3 (2.1 × 100 mm, 1.8 µm) (Waters, Milford, MA, USA). The column was maintained at 40 °C. The flow rate and injection volume were set at 0.3 mL/min and 2 μL, respectively. Mass spectrometric detection of metabolites was performed on Q Exactive Focus (Thermo Fisher Scientific, USA) with an ESI ion source. Simultaneous MS1 and MS/MS (Full MS-ddMS2 mode, data-dependent MS/MS) acquisition was used. The positive and negative ion modes were used to collect data, respectively, with a resolution of 70,000 for a first-level full scan with a scanning range of 100–1000 m/z, and HCD was used for second-level cracking with a collision energy of 30 eV and a second-level resolution of 17,500. The first 3 ions were broken up before the signal was collected, and dynamic exclusion was used to remove interference information. Public databases such as HMDB and Massbank were used for substance identification, and the metabolites were matched with the fragment ions of each metabolite in the database.

### Optimization of lignin degradation enzyme

QL-Z3 was cultivated in LB medium, then transferred 1% strain to the lignin culture medium. The main factors affecting lignin degradation enzyme activities include carbon source, nitrogen source, temperature pH, etc., which were optimized through variations of single factors and Orthogonal optimization.

## Results and discussion

### Strain characteristics

Lignin, cellulose, and hemicellulose constitute the whole biomass. At present, cellulose and hemicellulose can be converted into value-added useful products such as fuel ethanol and soluble sugars by enzymatic and/or fermentation pathways. To solve the difficulty of lignin degradation, reducing the cross-linking with other polymers in biomass is an important consideration in lignin biorefinery*. Erwinia billingiae* QL-Z3 was previously isolated from soil samples and stored in 25% glycerol at − 80 °C. It is known that the strain QL-Z3 can degrade lignin at 30 °C. [21]. Now we use scanning electron microscopy (SEM) imaging to reveal that QL-Z3 was a typical bacilliform with a rough surface and irregular folding of the cell surface (Additional file 1: Fig. S1). *Erwinia* was reported to be related to bacterial soft rot when it was first discovered and named [29], most studies focus on the pathogenic mechanism of plants at present. *Erwinia billingiae* QL-Z3 is the only *Erwinia* strain that had been confirmed to degrade lignin, while more researchers pay attention to *Bacillus*, *Pseudomonas* and other lignin-degrading strains.

### Influence of culture conditions on lignin degradation

While ligninolytic microbial species have been isolated and identified in recent years, there were also reports on the effects of culture conditions on lignin degradation [30]. The newly isolated *E. billingiae* QL-Z3 was capable of using alkali lignin, a common kraft lignin that constitutes about 85% of the total world lignin production [31], as a sole carbon source. When pH and nitrogen source were fixed, varying alkali lignin concentrations affected lignin degradation (Additional file 1: Fig. S2). Increasing lignin concentration from 0.5 to 1.5 g/L enhanced lignin degradation from 14.5% to 18.3%, respectively, while further increasing lignin concentration to 2.0 g/L reduced lignin degradation to 11.8%.

In addition to carbon source, nitrogen source has also been shown to play a role in lignin biodegradation. Supplementing nitrogen increased lignin degradation by the bacterium *Rhodococcus opacus* PD630 by about 10% [32], but did not significantly influence lignin degradation by the fungus *Phanerochaete chrysosporium* [33]. In this study, we found that ammonium sulphate ((NH_4_)_2_SO_4_) supplemented medium resulted in onefold higher lignin weight loss (~ 20%) than ammonium nitrate (NH_4_NO_3_), sodium nitrate (NaNO_3_), or tryptone supplemented medium (~ 10%). pH was another culture condition that showed significant influence on lignin degradation by QL-Z3, which was over 50% higher at acidic or neutral pH than basic pH (Additional file 1: Fig. S2).

**Fig. 1:**
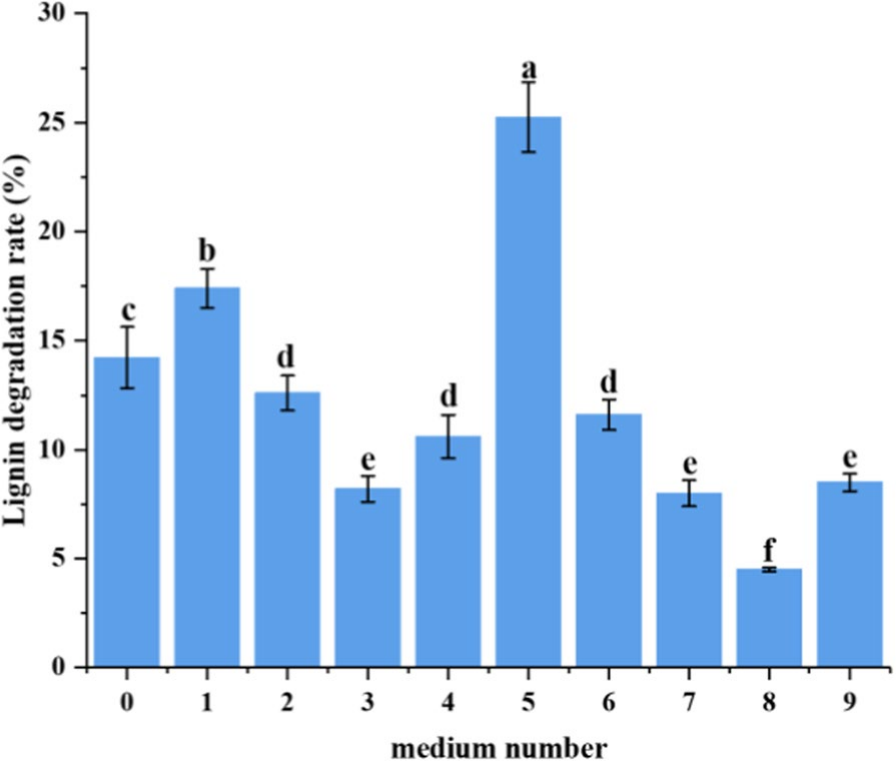
Effect of three factors on lignin degradation rate of *E. billingiae* QL-Z3. Medium Number: 0, 2.0 g/L lignin, pH = 7, tryptone (initial medium); 1, 1.0 g/L lignin, pH = 7, (NH_4_)_2_SO_4_; 2, 1.0 g/L lignin, PH = 5, NaNO_3_; 3, 1.0 g/L lignin, pH = 9, tryptone; 4, 1.5 g/L lignin, pH = 9, NaNO_3_;5, 1.5 g/L lignin, pH = 5, (NH_4_)_2_SO_4_; 6, 1.5 g/L lignin, pH = 7, tryptone; 7, 2.0 g/L lignin, pH = 5, tryptone; 8, 2.0 g/L lignin, pH = 7, NaNO_3_; 9, 2.0 g/L lignin, pH = 9, (NH_4_)_2_SO_4_. Average values of three replicates are shown with the standard errors of the mean shown as error bars. Each experimental group was conducted in triplicate

Based on the lignin degradation rates at different single factor optimization of each gradient, the top three conditions (lignin concentration, pH, nitrogen source) and three gradients were chosen for further orthogonal experiments. The optimal results are shown in Fig. 1, the orthogonal experimental listed in Additional file 1: Table S1-1 and Additional file 1: Table S1-2, respectively. Compared with the initial medium (medium 0 in Fig. 1), when the lignin concentration was fixed at 1.5 g/L, pH = 5, nitrogen source was (NH_4_)_2_SO_4_, the optimized medium enhanced the degradation rate from 14.23% to 24.25%, which equates to an increase of 41.32%. Compared with other lignin-degrading strains (Additional file 1: Table S2), QL-Z3 has the advantages of short culture cycle, simple fermentation conditions and higher degradation ability.

### Genomic analysis and functional verification

#### Genomic identification of candidate ligninolytic genes and expression analysis

To further dissect functional genes for lignin degradation in QL-Z3 via genome sequencing. As shown in Additional file 1: Fig. S3, the complete genome of QL-Z3 consisted of a circular chromosome (4286,943 bp) and a circular plasmid (526,446 bp), whose GenBank accession numbers were CP037949 and CP037950, respectively. The G + C content of the chromosome and the plasmid were 56.25% and 54.67%, respectively (Additional file 1: Table S3). The chromosome contained 4556 predicted coding sequences (CDSs), 22 rRNA genes (8 5S rRNA, 7 16S rRNA, 7 23S rRNA), and 78 tRNA genes, while the plasmid contained 166 predicted CDSs. Among these CDSs, 4428 chromosome genes and 140 plasmid genes were predicted to encode proteins through the Non-Redundant Protein Sequence (NR) Database. 3445 genes can be classified into 25 functional categories based on clusters of orthologous groups (COG) designations, including those of intracellular trafficking, secretion and vesicle transport, signal transduction mechanisms, carbohydrate transport and metabolism (Additional file 1: Fig. S4). Genome analysis based on KEGG database identified about 287, 146, and 432 genes that are potentially involved in membrane transport, signal transduction, and carbohydrate metabolism, respectively; 74 genes belong to Xenobiotics biodegradation and Metabolism class, including glutathione S-transferase (GST), acylphosphatase which are related to Aminobenzoate degradation, Metabolism of xenobiotics by cytochrome P450 (Additional file 1: Fig. S5). Genome analysis based on Carbohydrate-Active enZYmes Database (CAZy database) further identified the CAZyme genes, including 44 glycosyltransferases (GTs), 54 glycoside hydrolases (GHs), 24 carbohydrate esterases (CEs), 16 auxiliary activities (AAs), 4 carbohydrate-binding modules (CBMs), and 1 polysaccharide lyase (GL) (Additional file 1: Fig. S6). The abundant GH, CBM and AA in QL-Z3 indicated that the strain also had potential cellulase, hemicellulase and pectinase, among which the AA9 family gene encoding Lytic polysaccharide monooxygenases had been confirmed to be involved in the degradation of lignocellulose [34].

To identify these enzymes related to lignin degradation by QL-Z3, the encoding genes known as lignin enzymes of lignin-degrading model strains *Sphingobium* sp. SYK-6, *Pseudomonas putida* KT2440 and other reported strains were selected as query sequences, and a BLASTp (Basic Local Alignment Search Tool) search of the QL-Z3 genome was carried out [16, 35, 36]. Then the reported enzymes in SYK-6 and KT2440 were compared with candidate lignin-degrading enzymes of QL-Z3 by sequence similarity and protein domain analysis. 16 enzymes were found to belong to the same protein family with their similar proteins (Table 1), and 14 of them had the same protein domains, indicating that these genes encoding proteins may have similar functions and lignin products. These oxidoreductases including laccases, Dyp-type peroxidases, catalases, dioxygenases, monooxygenases, glutathione synthases, superoxide dismutases etc. Laccase can degrade lignin on its own through the coordinated transfer of electrons by four copper ions [37], and there are fewer examples of bacterial laccases than of fungal laccases, therefore, it is very necessary to find more bacterial laccases. Some peroxidase in lignin-degrading white-rot fungi belong to the plant peroxidase superfamily (Class II), but LiP, MnP seem to be limited to fungi, which is rarely found in bacteria. Bacteria are relatively rich in other types of peroxidases, including Dyp peroxidase and Mn-catalase [38]. Superoxide dismutase is an antioxidant enzyme that can degrade different lignin model substrates into several compounds and play a critical role in the polymerization of lignin monomers [39, 40]. In addition, enzymes such as dioxygenase, monooxygenase, and glutathione synthase may also degrade lignin through REDOX action. Xu et al. [35] have already identified these enzymes might be involved in the lignin catabolism pathway.

**Table 1:** Similarity analysis of protein domain and lignin products comparison in candidate lignin degrading genes

Gene ID	Gene name	Encode protein	Reference organism	Same domain	% Identity/aa	Accession no	Lignin products
Chr_48	/	Dyp-type peroxidase	*Pseudomonas putida* KT2440 *Streptomyces pharetrae* CZA14	IPR048328	31/298 25/298	WP_010954130 OSZ61955	Vanillic acid
Chr_205	pgeF	polyphenol oxidase/Laccase	*Pseudomonas putida* KT2440 *Sphingobium* sp. SYK-6	–	56/242 35/242	WP_010951886 WP_014078201	/
Chr_410	gshB	Glutathione synthase	*Pseudomonas putida* KT2440 *Sphingobium* sp. SYK-6	IPR004215 IPR011761 IPR004218	66/314 42/314	WP_010955561 BAK68397	/
Chr_468	/	SDR family oxidoreductase	*Pseudomonas putida* KT2440 *Sphingobium* sp. SYK-6	/	33/250 38/250	WP_003249597 WP_014077645	/
Chr_858	tauD	Taurine dioxygenase	*Sphingobium* sp. SYK-6	IPR003819	45/284	WP_014075385	/
Chr_996	/	NADP dependent oxidoreductase	*Pseudomonas putida* KT2440 *Sphingobium* sp. SYK-6	IPR006115 IPR029154	31/304 31/304	WP_010955321 WP_014076334	/
Chr_1016	gorA	Glutathione-disulfide reductase	*Pseudomonas putida* KT2440 *Sphingobium* sp. SYK-6	IPR023753 IPR004099	29/450 39/450	WP_010953139 WP_014076796	/
Chr_1236	sodA	Superoxide dismutase	*Pseudomonas putida* KT2440 *Sphingobacterium* sp. T2	IPR019831 IPR019832	57/205 52/205	AAN66571 WP_231561313	Benzoic acid /vanillin/catechin
Chr_1594	/	Quinone oxidoreductase	*Pseudomonas putida* KT2440 *Sphingobium* sp. SYK-6	IPR013154 IPR013149 IPR020843	30/326 31/326	WP_010951478 WP_014077905	/
Chr_2803	ssuD	Alkanesulfonate monooxygenase	*Pseudomonas putida* KT2440	IPR011251	80/381	WP_003255833	/
Chr_3132	katE	Catalase	*Pseudomonas putida* KT2440 *Streptomyces* sp. UNC401CLCol	IPR010582 IPR011614	41/754 42/754	WP_010951777 WP_028959332	/
Chr_3182	gloA	Lactoylglutathione lyase	*Pseudomonas putida* KT2440	IPR004360 IPR037523	34/135	WP_010954597	/
Chr_3330	ssuD	Alkanesulfonate monooxygenase	*Pseudomonas putida* KT2440	IPR011251	62/388	WP_003255833	/
Chr_3467	/	Catalase	*Pseudomonas putida* KT2440 *Streptomyces* sp. UNC401CLCol	IPR010582 IPR011614	53/489 52/489	WP_010951777 WP_028959332	/
Chr_3587	/	Manganese catalase	*Sphingobium* sp. SYK-6	IPR039377	66/305	WP_014075027	/
Chr_4287	/	Catalase	*Pseudomonas putida* KT2440 *Streptomyces* sp. UNC401CLCol	IPR010582 IPR011614	60/479 55/479	WP_010951777 WP_028959332	/

“–” means protein domain is not same, “/” means domain or lignin products were unknown [16, 35, 36]

**Fig. 2:**
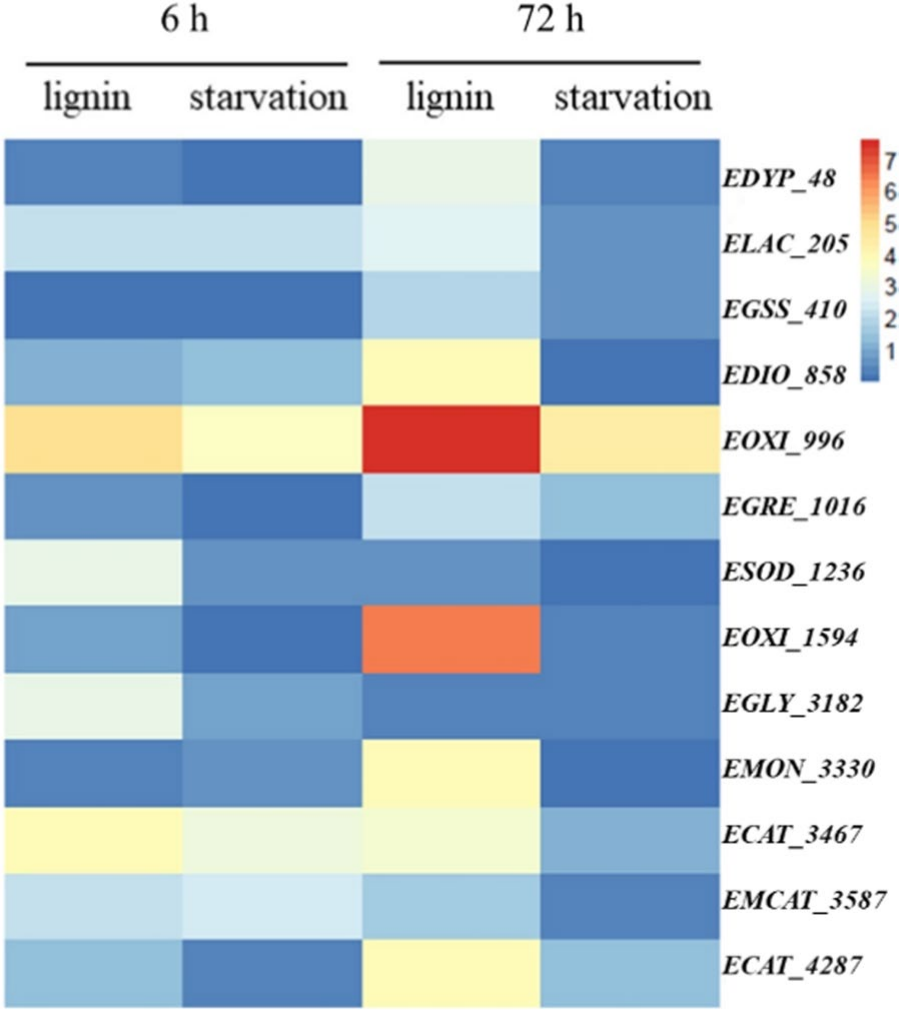
Gene real-time expression levels at 6 h and 72 h under lignin and starvation Collected Ct values of lignin, glucose and starvation group of different genes at 6 h and 72 h, then analyzed and calculated the relative expression levels of corresponding target genes in different samples. Finally, the expression level of genes in the glucose group was set as the control value 1, the relative expression levels of the lignin and starvation group were calculated, respectively, when glucose was used as the control, and then Log_2_E logarithm was used to draw the heat map. Each experimental group was conducted in triplicate

To confirm the expression of the 16 candidate ligninolytic genes are subjected to induction by lignin or carbon starvation, RT-qPCR was applied to the samples grown in the media supplemented with or without 1.5 g/L lignin for 6 h and 72 h. The results showed that the transcription levels of the 3 genes (*Chr_468, Chr_2803, Chr_3132*) were not regulated by lignin (Additional file 1: Table S6), 13 genes were identified to be induced by lignin, including Dyp-type peroxidase (*EDYP_48*), laccase (*ELAC_205*), glutathione synthase (*EGSS_410*), dioxygenase (*EDIO_858*), oxidoreductase (*EOXI_996*, *EOXI_1594*), glutathione reductase (*EGRE_1016*), superoxide dismutase (*ESOD_1236*), glutathione lyase (*EGLY_3182*), monooxygenase (*EMON_3330*), catalase (*ECAT_3467*, *ECAT_4287*), and manganese catalase (*EMCAT_3587*) (Fig. 2). The results revealed that *ELAC_205*, *EOXI_996*, *ESOD_1236*, *EGLY_3182*, and *ECAT_3467*, *EMCAT_3587*, *ECAT_4287* were involved in transcription at 6 h, which may have the function of the LMEs to convert lignin into smaller aromatics that can be imported into the cell for aromatic catabolism [17, 41]. Interestingly, *ELAC_205, ECAT_3467* and *EMCAT_3587* had high responses in both lignin and starvation medium at the first 6 h, indicating that the three genes were also induced by starvation response. Such starvation response gradually decreased and have more regulated by lignin after 72 h. We notice that *ELAC_205* has no domain predicted results, but it belongs to the same family in *Pseudomonas putida* KT2440 and *Sphingobium* sp. SYK-6, which contains four classical copper ion binding sites, may catalyze the oxidation of various phenolic substrates via the reduction of oxygen to water. The highly abundant catalase and manganese catalase in QL-Z3 are catabolic genes that may be involved in lignin depolymerization or oxidative stress reaction [34]. We speculate that *ECAT_3467*, *EMCAT_3587* and *ECAT_4287* can not only provide “H” and “O” atoms for the decomposition reaction as a substrate, but also play a vital role in aromatic catabolic reactions. *ECAT_3587* contains an extraordinary manganese catalase type domain, that is, the active site containing manganese ions, which can oxidize Mn^2+^ to Mn^3+^, and Mn^3+^ can oxidize a large number of phenolic substrates. It has been reported that superoxide dismutase has lignin-degrading activity in *Sphingobacterium* sp. T2 [39], Lin L found through proteomic analysis that when lignin is used as a carbon substrate, the encoded superoxide dismutase is expressed extracellular, indicating its potential role in lignin decomposition [42]. Similar to their results, *ESOD_1236* have highly expression level at 6 h, while it decreased at 72 h. Moreover, *EGLY_3182* only participated in the regulation at 6 h, glutathione reductases from *Sphingobium* sp. SYK-6 (LigE and LigF) were reported they have the ability to cleave the intermediate with the attachment of glutathione at the C position [20, 43], but *EGLY_3182* has no similar domain with them.

*EDYP_48*, *EDIO_858*, *EOXI_996*, *EOXI_1594*, *EMON_3330* were strongly induced by lignin at 72 h, indicating that they also played an important role in lignin degradation. Compared with them, *EGSS_410* and *EGRE_1016* expression levels were relatively lower. Dyp is a peroxidase involved in extracellular lignin depolymerization, Ahmad M [44] found that knockout of Dyp gene in *jostii Rhodococcus* RHA1 significantly reduced the ability of lignin depolymerization compared with the wild type. Dyp peroxidase can oxidize the β-O-4 bond of lignin in the presence of manganese ions to obtain the lignin product vanillic acid [35]. After the lignin macromolecule depolymerization, LDA enzymes assist the core lignin-degrading enzymes to degrade the lignin monomer. Monooxygenase (*EMON_3330*) and dioxygenase (*EDIO_858*) are involved in lignin degradation in other studies [45, 46], we believe that they should be considered as LDA enzymes. In addition, qPCR results identified the highly expression of oxidoreductases (especially EOXI_996) indicating its potential role in lignin decomposition, but the mechanisms of *EOXI_996* and *EOXI_1594*, oxidoreductase proteins, in the oxidative decomposition of lignin are unknown. In summary, we select eight different types of genes who have expression at 72 h (*EDYP_48*, *ELAC_205*, *EDIO_858*, *EOXI_996*, *ESOD_1236*, *EMON_3330*, *ECAT_3467*, *EMCAT_3587*) to verify the function via gene knockout and heterologous expression in the next step.

#### Gene knock-out and phenotypic validation

Deletion of *EDYP_48*, *ELAC_205*, *EDIO_858*, *EOXI_996*, *ESOD_1236*, *EMON_3330*, *ECAT_3467* and *EMCAT_3587* were confirmed using PCR amplification of the 8 target genes (Additional file 1: Fig. S7). Complementation of the deleted genes was confirmed using the replenishment plasmid pKT100 with universal primer F/R (Additional file 1: Fig. S8).

**Fig. 3:**
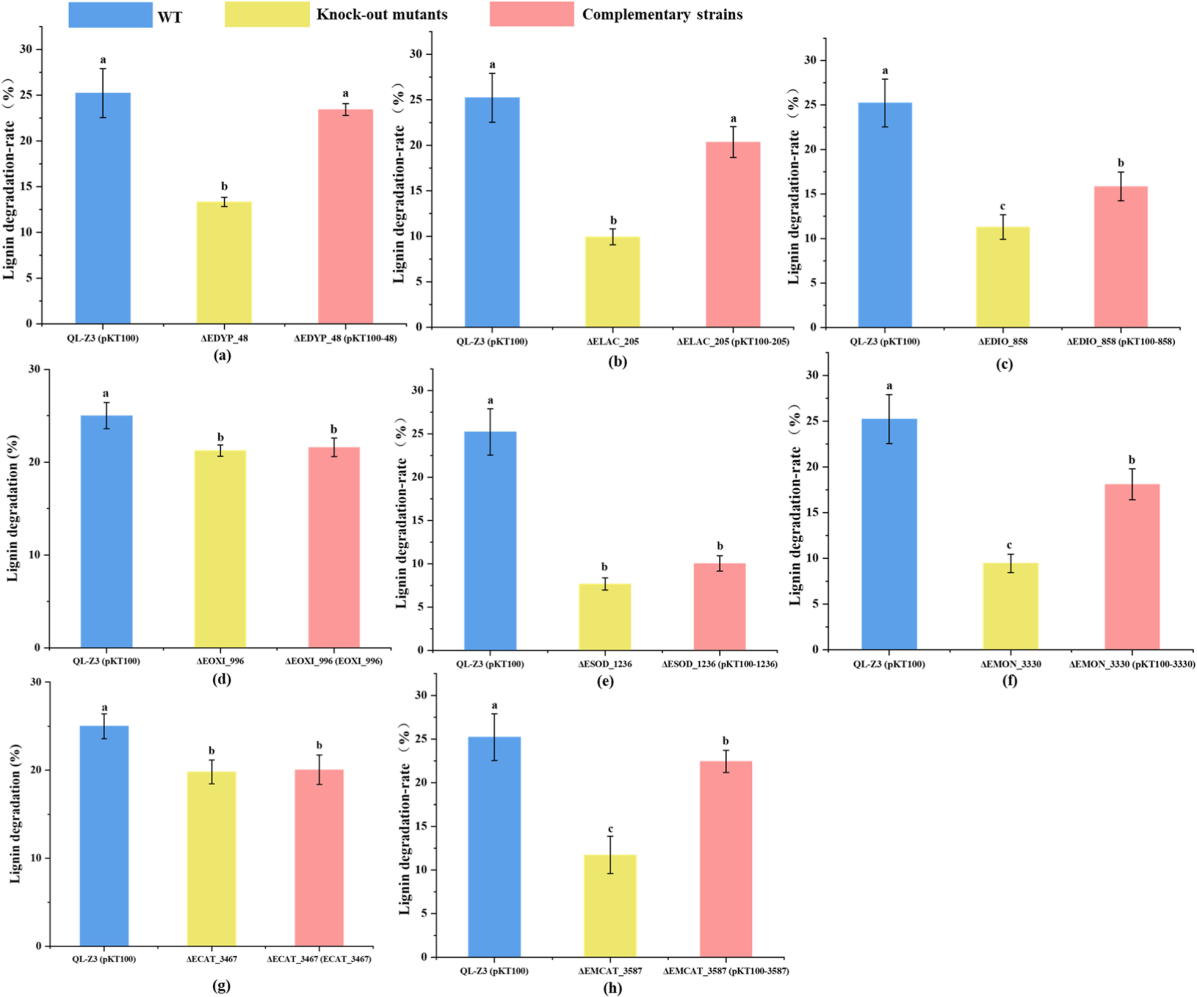
Determination of lignin degradation rate of knock-out mutant mutants and complementary strains in strain QL-Z3. Blue: wild type (WT), yellow: knock-out mutants, red: complementary strains. **a** *EDYP_48* wild type, knock-out mutant and complementary strain; **b** *ELAC_205* wild type, knock-out mutant and complementary strain; **c** *EDIO_858* wild type, knock-out mutant and complementary strain; **d** *EOXI_996* wild type, knock-out mutant and complementary strain; **e** *ESOD_1236* wild type, knock-out mutant and complementary strain; **f** *EMON_3330* wild type, knock-out mutant and complementary strain; **g** ECAT_3467 wild type, knock-out mutant and complementary strain; **h** *EMCAT_3587* wild type, knock-out mutant and complementary strain. a, b, c: significant difference (*p ≤ 0.05) (ANOVA analysis). Average values of three replicates are shown with the standard errors of the mean shown as error bars. Each experimental group was conducted in triplicate

**Fig. 4:**
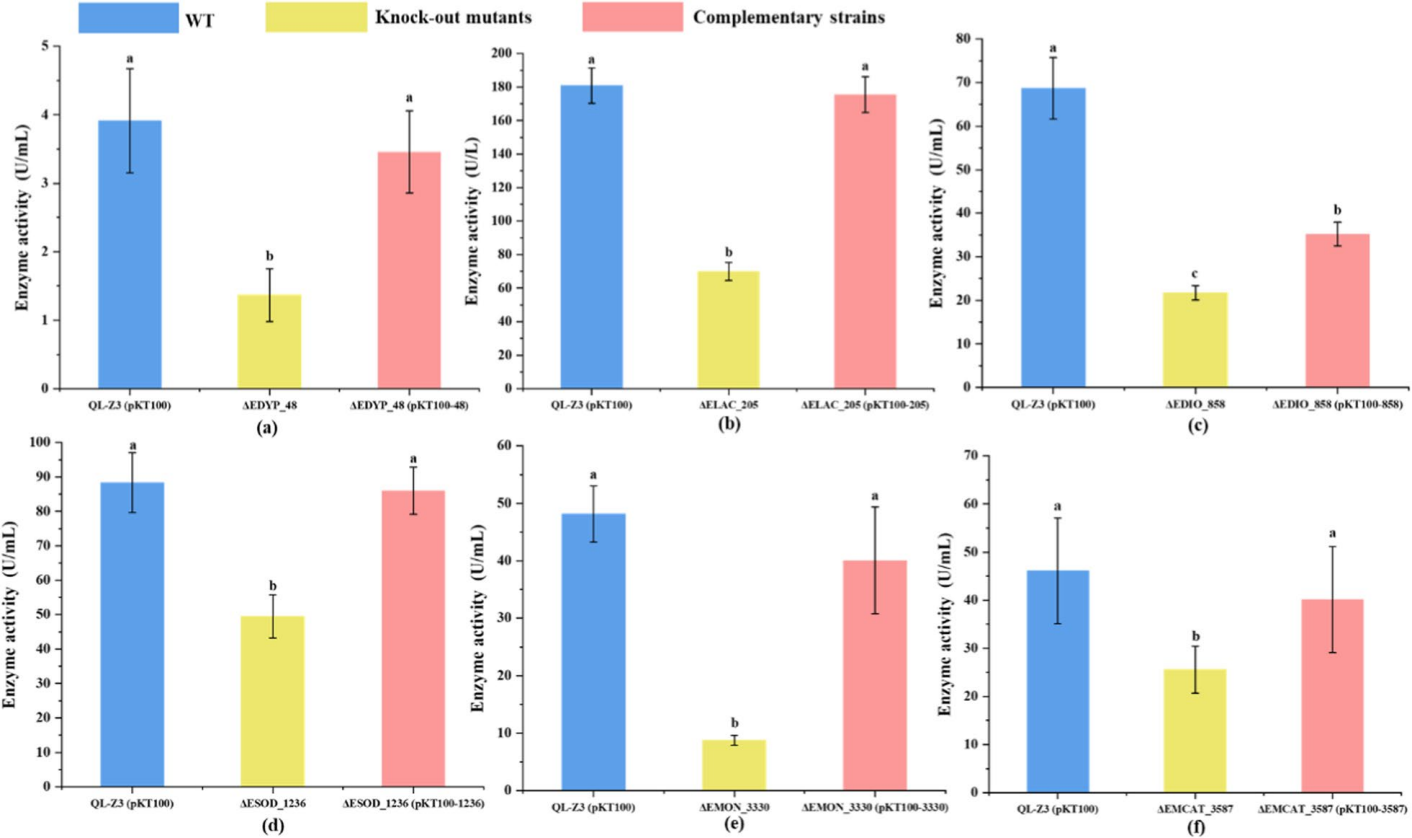
Enzyme activity of knock-out mutant mutants and complementary strains in strain QL-Z3. Blue: wild type (WT), yellow: knock-out mutants, red: complementary strains. **a** *EDYP_48* wild type, knock-out mutant and complementary strains; **b** *ELAC_205* wild type, knock-out mutant and complementary strains; **c** *EDIO_858* wild type, knock-out mutant and complementary strains; **d** *ESOD_1236* wild type, knock-out mutant and complementary strains; **e** *EMON_3330* wild type, knock-out mutant and complementary strains; **f** *EMCAT_3587* wild type, knock-out mutant and complementary strains. a, b, c: significant difference (*p ≤ 0.05) (ANOVA analysis). Average values of three replicates are shown with the standard errors of the mean shown as error bars. Each experimental group was conducted in triplicate

Then, the degradation rate and enzyme activity of the knock-out mutants and the complementary strains were verified. As shown in Fig. 3, After 72 h cultivation, lignin degradation by the wild-type strain reached about 25%. Among the mutants with gene deletions *ΔEDYP_48*, *ΔELAC_205*, *ΔEDIO_858*, *ΔEOXI_996*, *ΔESOD_1236*, *ΔEMON_3330*, *ΔECAT_3467* and *ΔECAT_3587* reduced lignin degradation rates of 11.65%, 16.73%, 13.71%, 3.67%, 17.30%, 15.55%, 5.95% and 8.94% were observed. Genetic complementation analysis demonstrated that complementary strains with six corresponding genes fully or partially restored the lignin degradation activities of the mutant strains (Fig. 3). The degradation rates of the two genes (*EOXI_996* and *ECAT_3467*) knock-out strains reduced less and did not differ significantly from those of their complementary strains (Fig. 3), that’s may because QL-Z3 contained many catalases and oxidoreductase which have the same function, while have generated other compensation pathways to complete the complex degradation process. Then we measured enzyme activities of six knock-out mutants and complementary strains, six enzyme activities assay further demonstrated that deletion of the ligninolytic genes resulted in a significant decrease in the activities of secreted ligninolytic enzymes, while expression of complementary genes fully or partially restored the decreased activities of extracellular ligninolytic enzymes (Fig. 4). Taken together, the results corroborate multiple enzymes in lignin biodegradation and synergism of different ligninolytic enzymes from QL-Z3.

#### FTIR analysis

**Fig. 5:**
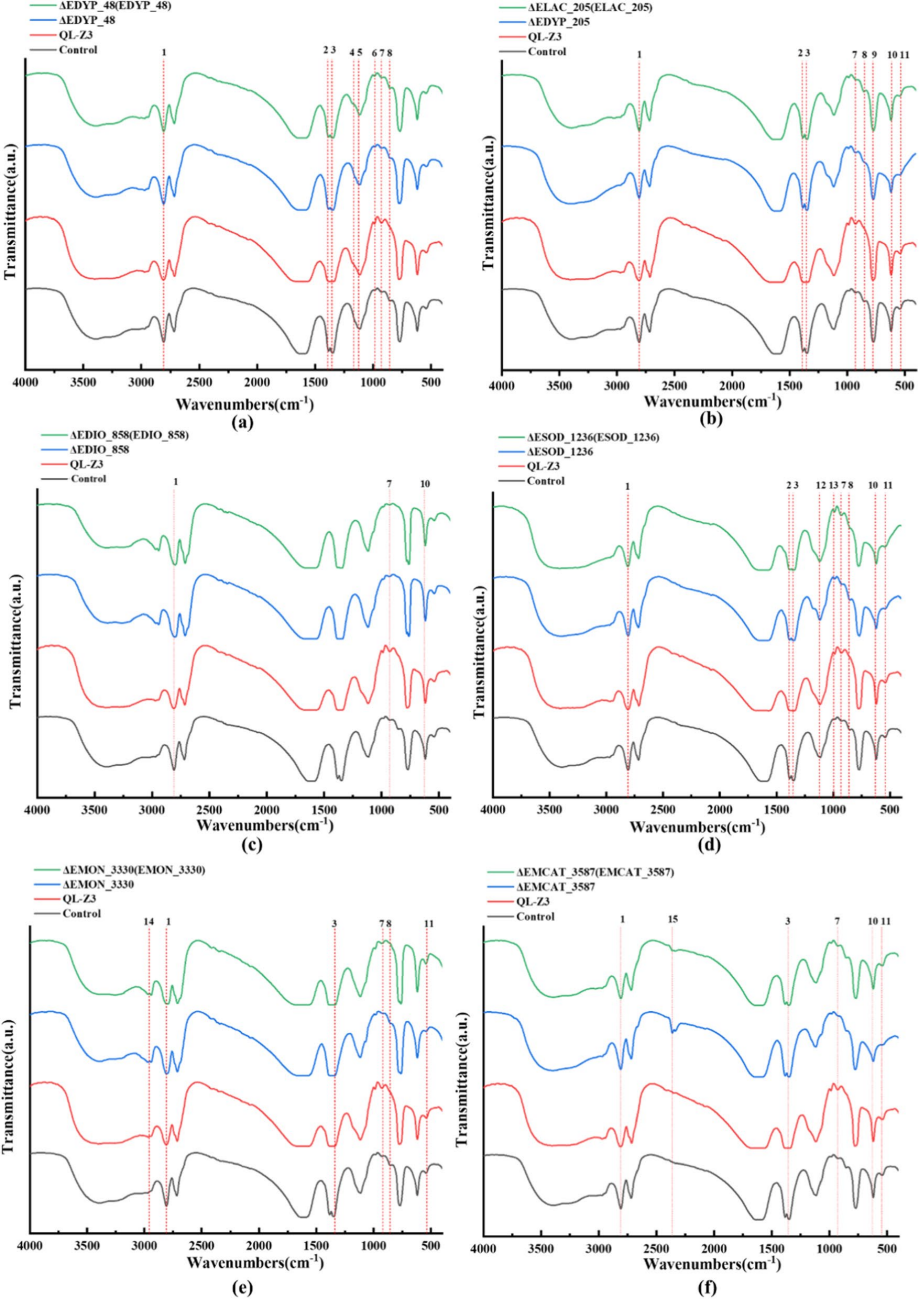
Fourier transform infrared spectra of alkali lignin degradation genes. **a** *EDYP_48*, **b** *ELAC_205*, **c** *EDIO_858*, **d** *ESOD_1236*, **e** *EMON_3330*, **f** *EMCAT_3587* in knock-out mutants (blue), complementary strains (green), wild type (WT, red) and alkali lignin without strains (black). Each experimental group was conducted in triplicate

The peaks in the FTIR spectra in lignin were identified by reference to the literature [47, 48], and the infrared spectra before and after degradation of alkali lignin by wild type, mutant type, and compensatory type (using non-inoculated strains as control) were analyzed. It was observed that the infrared spectral intensity of alkaline lignin changed significantly at 2800 cm^−1^, 1700 cm^−1^–1000 cm^−1^ (lignin fingerprint), and 1000 cm^−1^–500 cm^−1^ bands with QL-Z3 treatment (Fig. 5). The absorption peak at 2800 cm^−1^ represents the antisymmetric stretching vibration of methyl group C–H bond and methoxy O–CH_3_ bond in lignin, and the strength of this peak decreases, indicating that lignin was degraded. In the wild type, the C–H deformation of the representative methyl group and the 1464 cm^−1^–1462 cm^−1^ cm vibration of the aromatic ring are reduced, which means that the content of the methoxy group is reduced due to demethylation [49]. Interestingly, with the exception of *EDIO_858* and *EMON_3330*, there was no significant difference between the complement and mutants of other genes and the control group, indicating that these genes were involved in the C–H bending of methyl groups, which caused strong shaking of the lignin skeleton. The intensity of the peak corresponding to the C–O bond (1147 cm^−1^) in lignin and the guaiac-based C–H plane (989 cm^−1^–846 cm^−1^) were changed, and the peaks of the wild type were obviously stronger, while the peak signal of the mutant type was weakened and disappeared, indicating that these genes played an important role in C–O and C–H fracture. In addition, the changes in the peaks of 623 cm^−1^ and 549 cm^−1^ also indicate that the C–H bond in the lignin skeleton has a violent oscillation, which is higher than that of mutant and complement type.

### Heterologous expression and characterization of ligninolytic enzymes

To further characterize the function of the ligninolytic enzymes encoded by genes from *E. billingiae* QL-Z3, recombinant plasmids expressing these proteins were constructed and transformed into *E. coli* BL21. Single colony grown on the culture dish was verified by colony PCR. Recombinant enzymes were induced by isopropyl-β-D-thiogalactopyranoside (IPTG) and purified by Ni–NTA affinity chromatography (Additional file 1: Fig. S9). The activities of these proteins were performed as described in Materials and Methods by using different substrates, such as H_2_O_2_ and veratryl alcohol etc. Enzyme activity assays of purified proteins demonstrated that as predicted by genomic and genetic analyses, recombinant proteins of EDYP_48, ELAC_205, EDIO_858, ESOD_1236, EMON_3330, and EMCAT_3587 possessed the activities of dye decolorization peroxidase, laccase, dioxygenase, superoxide dismutase activity, monooxygenase, and manganese catalase, respectively (Additional file 1: Table S7).

After comprehensive analyses, including bioinformatics analysis, RT-qPCR, genetics, phenotyping, biochemistry, and FTIR, we believed that enzymes encoded by *EDYP_48*, *ELAC_205*, and *ESOD_1236* have an important role in the first step of lignin depolymerization process. Therefore, we investigated their enzymatic characteristics. The degradation efficiency of 2, 2’-azino-bis (3-ethylbenzothiazoline-6-sulfonic acid) (ABTS, lignin structure analogs) by these three enzymes were tested. After the assays, the resulting visible ESOD_1236 had weak oxidation activity on ABTS, which was consistent with the results reported that SOD treatment did not produce precipitation in acid-precipitable polymeric lignin [42]. These results suggested that SOD was an LDA enzyme and had a poor ability to degrade lignin alone.

The activity conditions (pH, temperature) of enzymes encoded by *EDYP_48*, *ELAC_205* were optimized (Additional file 1: Fig. S10), and the activity of ELAC_205 was measured in sodium acetate buffer at pH 0.5 to 8.0. The results showed that ELAC_205 had the highest enzyme activity in sodium acetate buffer at pH and temperature was 1.0 and 50 °C, respectively, which had excellent tolerance in strong acids. Because of the partial denaturation of EDYP_48 at pH < 4.0, we measured EDYP_48 activity only in sodium acetate buffers at pH 4.0 to 9.0. The results showed that EDYP_48 had the highest enzyme activity at 40 °C and pH 5.0. On this basis, EDYP_48 (Km = 0.73 mM) and ELAC_205 (Km = 0.36 mM) both showed catalytic activity with substrate ABTS. Compared with reported proteins, it was observed that EDYP_48 performed higher catalytic efficiency (Table 2).

**Table 2:** Kinetics parameters comparison of EDYP_48 and ELAC_205

Name	Encoding protein	Km (mM)	Kcat (s^−1^)	Kcat/Km (s^−1^mM^−1^)	References
EDYP_48	Dyp-type peroxidase	0.730 ± 0.021	5.87	8.04 × 10^3^	This study
PputA514_2985	Dyp-type peroxidase	0.585 ± 0.012	4.85	8.29 × 10^3^	[42]
PputA514_3152	Dyp-type peroxidase	0.217 ± 0.022	1.42	6.53 × 10^3^	[42]
ELAC_205	Laccase	0.360 ± 0.07	0.60	1.67 × 10^3^	This study
LacZ1	Laccase	0.35	/	/	[37]
Lacc	Laccase	1.44	1378.09	9.54 × 10^5^	[50]

### Enzymatic hydrolysis and product analysis of lignin by ligninolytic enzymes

#### Enzymatic hydrolysis rate of alkali lignin

**Fig. 6:**
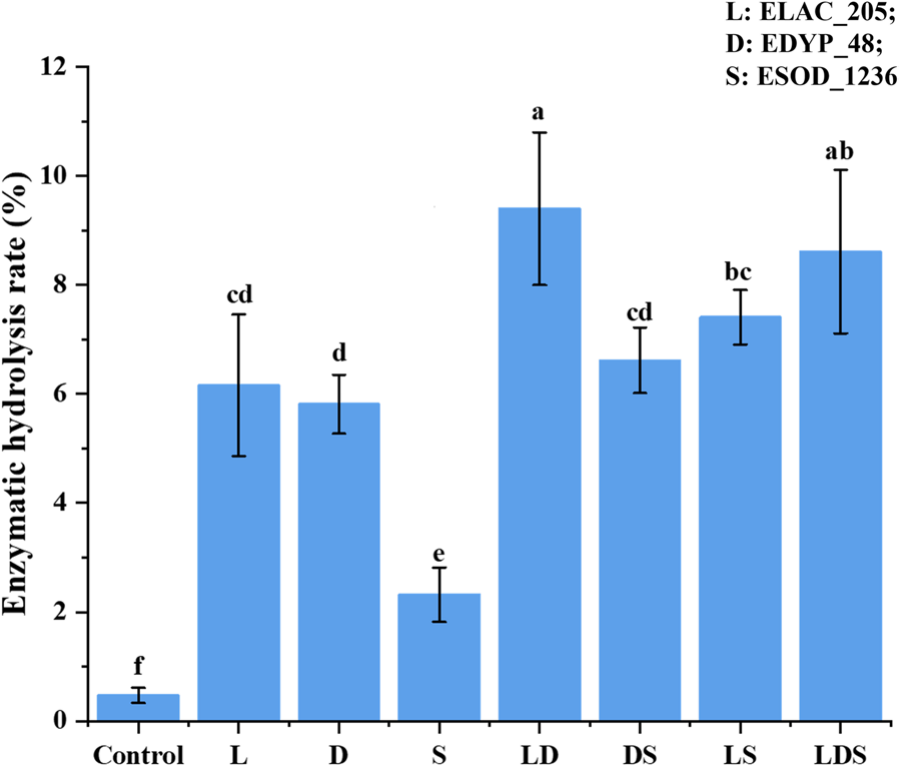
Enzymatic hydrolysis rate of alkali lignin in different combinations of the three enzymes. L: ELAC_205; D: EDYP_48; S: ESOD_1236; LD: ELAC_205 and EDYP_48; DS: EDYP_48 and ESOD_1236; LS: ELAC_205 and ESOD_1236; LDS: three enzymes mixed. a, b, c: significant difference (*p ≤ 0.05) (ANOVA analysis). Average values of three replicates are shown with the standard errors of the mean shown as error bars. Each experimental group was conducted in triplicate

EDYP_48 and ELAC_205 are the core LME enzymes in the process of lignin degradation. It is necessary to elucidate the degradation products of a single enzyme and the synergism of them. As an LDA enzyme, ESOD_1236 can also assist the LME enzyme in producing various centrosomes of lignin [39]. Then we calculated the enzymolysis efficiency of alkaline lignin under different combinations of three pure enzymes. When three enzymes were used alone (Fig. 6), the degradation rates of ELAC_205 (6.16%) and EDYP_48 (5.81%) were significantly higher than those of ESOD_1236 (2.32%). In the case of double enzyme combination, the efficiency of LD (ELAC_205 and EDYP_48) combination was the highest (9.40%), and there was no significant difference between LD combination and triple enzyme combination.

#### LC–MS determination and product analysis

**Fig. 7:**
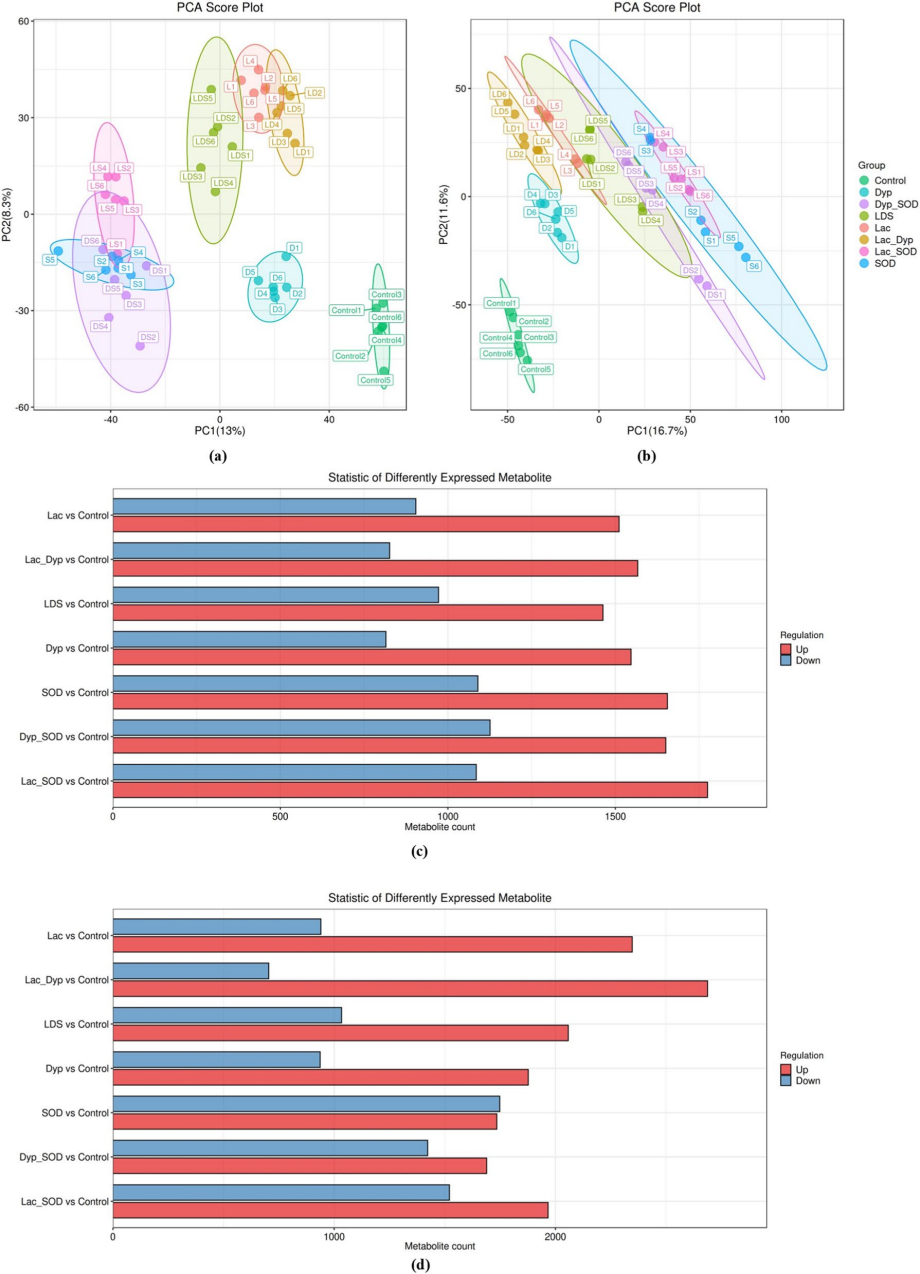
Three enzymes degrade alkali lignin in different combinations. **a** PCA scores with positive ion modes, **b** PCA scores with negative ion modes and **c** differential metabolite statistics with positive ion modes, **d** differential metabolite statistics with negative ion modes. Each experimental group was conducted in sextuplicate

To further know how three enzymes encoded by genes of QL-Z3 depolymerize large lignin polymers to produce phenoxy radical intermediates and then the conversion of heterogeneous lignin derivatives to central intermediates. The metabolites of three enzymes, in different combinations were detected via LC–MS. The metabolites were identified by accuracy mass and MS/MS data which were matched with the metabolite database. The changes of each degradation product were obtained by quantitative comparison with the peak area of the control group, the significant difference analysis and screening were conducted from the list of primary and secondary metabolites (Fig. 7). PCA score plot showed obvious separation of different treatments, especially the control group and Dyp group, which indicated significant changes of metabolites after enzymolysis for 24 h, and the amount of “up” “down” metabolites varies with different treatments.

According to LC–MS analysis, the degradation products produced by enzymatic hydrolysis of alkali lignin were mainly acids, alcohols, ketones, phenols and aromatics. Compared with the control group, polyaromatic acids were listed in Additional file 1: Table S8, which significantly up-regulated and down-regulated as degradation products. Single enzyme hydrolysis of alkali lignin by Lac produced the most hydrolysis products, a total of 36 up regulation and 16 down regulation, the contents of the most including phenylethanol, protocatechuic acid, catechin, and benzoic acid, because laccase is generally considered to play an important role in the ring opening of benzene, and studies have shown that it can bind to Cu^2+^ ions and catalyze oxidation reactions including polymer degradation and oxidative coupling of phenolic compounds to generate more degradation products [37].

Dyp-peroxidase recently received attention due to its special primary and tertiary structure and broad substrate specificity, which has a higher REDOX potential without additional mediator to promote lignin degradation [51]. Rahmanpour et al. [52] found that overexpression of DyP1B in *E. coli* has the activity of oxidizing Mn^2+^ and degrading powdery wheat grass lignocellulose. It has been reported that the DyP peroxidase of *Basidiomycete Auricularia auriculajudae* has the potential of cracking lignin substructural bonds [53], EDYP_48 seems to have the same potential. Lignin degradation by EDYP_48 alone accumulated 32 main products and consumed 12 products. Similar to LiP mechanism, EDYP_48 utilize free radical reaction to destroy the C_α_–C_β_ bond on the monomer side chain of lignin [35], thus producing a large amount of methoxyphenol and its derivatives. In addition, synergy of EDYP_48 and ELAC_205 produced the largest variety of “up” products, such as aromatic acids and quinones (Additional file 1: Table S8).

The treatment of organic solutes lignin by MnSOD1&2 would result in aryl-Cα oxidation bond cleavage, as well as C_α_–C_β_ oxidation cleavage, decarboxylation and O-demethylation, and authors speculated that the reaction cycle consisted of two half reactions, that is, Mn (III) oxidized superoxide to dioxygen, and Mn (II) reduced superoxide to hydrogen peroxide [39]. In this study, the products of ESOD_1236 were the least in several combinations, but increased after combining with the two enzymes. In the aromatic degradation products obtained by SOD alone, products such as 1-Naphthol, 2-Ketobutyric acid, o-Xylene, alpha-Ketoisovaleric acid, Aminomalonic acid, (+)-Syringaresinol, and 3-Furoic acid intermediates, however, these intermediates were not detected in L (ELAC_205) and D (EDYP_48) alone or the combined of the two and three enzymes, indicating that further degradation of these intermediates by SOD is difficult. We speculated that the large lignin polymer was depolymerized under the first step of hydrolysis/LME. Lac generates phenoxy free radicals through the synergistic action of Dyp peroxidase under aerobic conditions, and then SOD and LME enzyme were co-oxidized and decomposed or demethylated to transform into more intermediates, forming an unstable aromatic ring structure and promoting the degradation of lignin.

#### Main degradation pathways of lignin by three enzymes

**Fig. 8:**
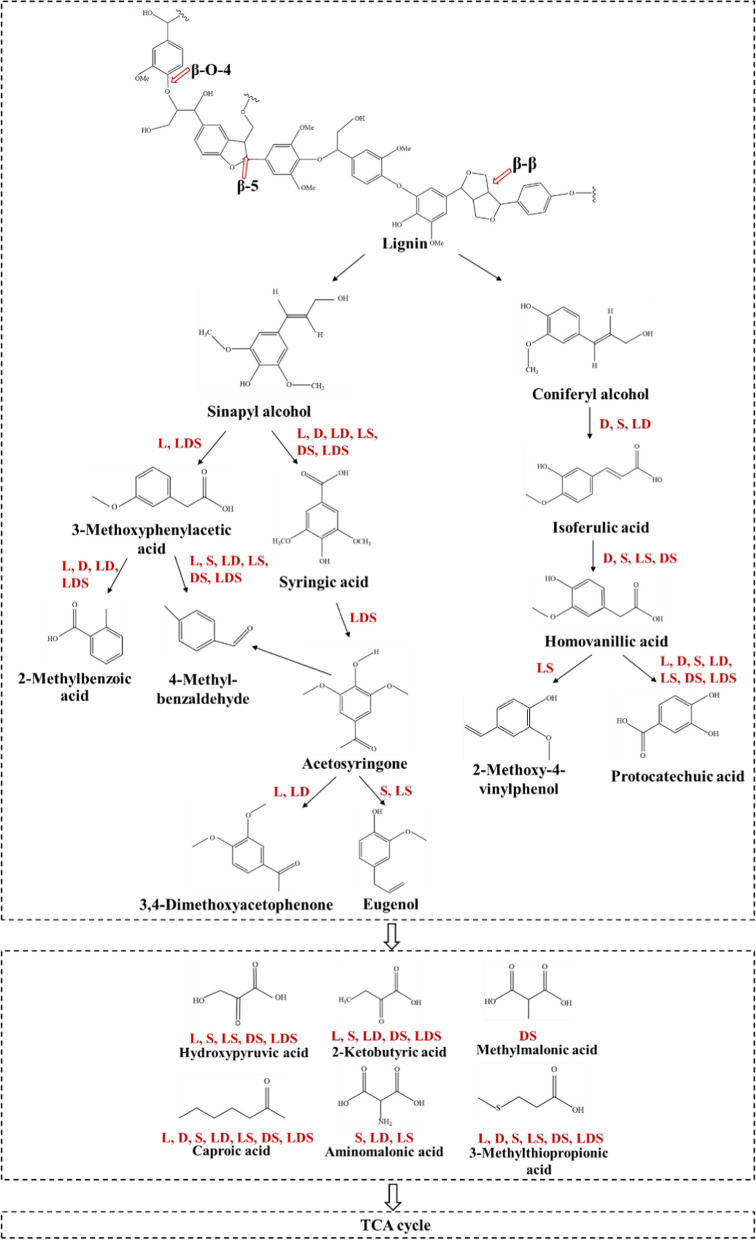
Proposed lignin degradation pathways with three enzymes. The enzymes final degradation products in the pathway map are identified via LC–MS and the reaction mechanisms are cross-validated by previous literature [39, 45–50, 52]. Red indicates that the products is produced in different combinations of the three enzymes. L: ELAC_205; D: EDYP_48; S: ESOD_1236; LD: ELAC_205 and EDYP_48; DS: EDYP_48 and ESOD_1236; LS: ELAC_205 and ESOD_1236; LDS: three enzymes mixed. Detailed enzyme information can be found in Additional file 1: Table S8

Lignin is formed from the guaiacyl units (G, precursor coniferol alcohol), the syringyl units (S, precursor sinapyl alcohol) and p-hydroxyphenyl units (H, precursor p-coumarol alcohol) by the polymerization of β-O-4, β-5 and β–β types. In this study, a comparison of the degradation products obtained from different combinations of EDYP_48, ELAC_205 and ESOD_1236, we found that sinapyl alcohol and coniferyl alcohol are the main monomer forms that exist after the first damage of alkaline lignin skeleton (Fig. 8). Next, under enzymatic catalysis, the phenylpropane monomer structure is converted into 3-Methoxyphenylacetic acid, 2-methoxyhydroquinone, syringic acid, 2, 4-dihydroxyacetophenone and other complex intermediates, which are often produced by the pyrolysis of C_α_–C_β_ phenols. Under aerobic conditions, 3-Methoxyphenylacetic acid reacts to form 2-Methylbenzoic acid and 4-Methylbenzaldehyde, syringic acid is demethylated to produce acetosyringone, which is reoxidized to produce eugenol and 3, 4-dimethoxyacetophenone. Ferulic acid is another phenolic component of lignocellulose, which was a significant product of coniferol monomer. In *Sphingomonas paucimobilis* SYK-6, ferulic acid coenzyme a converts ferulic acid to vanillin; Decarboxylase of *Bacillus* BP-7, which can be converted into corresponding 4-vinyl aromatic compounds [54]. In this study, the addition of ESOD_1236 down-regulated isoferulic acid and produced a variety of benzaldehyde and vanillic acid products. Vanillic acid is converted into protocatechuic acid by demethylation of three combined enzymes. Literature indicated that protocatechuic acid is mainly degraded by protocatechuic 3, 4-dioxygenase through oxidative ring cleavage, and then metabolized via the β-keto-adipic pathway or the orthogonal cleavage pathway [55], but LDS (three enzymes mixed) lacks this capability, which becoming a rate-limiting step and results in the accumulation of protocatechuic acid more than 1000 times. Finally, under the action of multi-enzyme oxidation, these intermediate aromatic rings break to form aliphatic compounds and enter the TCA cycle.

### Optimization of enzyme activities

Three extracellular enzyme activities were determined by UV–visible spectrophotometry at fixed absorbance. As is evident in Table 3, 2.0 g/L lignin concentration, pH = 9.5, nitrogen Source was NH_4_NO_3_, supported the highest enzyme activity of all. At 30 °C, Lac and MnP maximum activity reached 265 U/L and 202 U/L, followed by LiP was 110 U/L at 25 °C.

**Table 3:** Effect of different single factor on ligin degradation enzyme activity of QL-Z3

Condition	Gradient	Lip (U/L)	Lac (U/L)	Mnp (U/L)
Lignin concentration	0.5 g/L	141	38	150
	1.0 g/L	195	56	149
	1.5 g/L	226	58	225
	2.0 g/L	293	78	326
	2.5 g/L	240	64	271
pH	5	80	113	161
	6.5	112	129	163
	8	115	214	220
	9.5	130	226	224
	11	109	146	219
Nitrogen source	NaNO_3_	151	138	120
	NH_4_NO_3_	245	152	198
	NH_4_SO_4_	128	113	110
	Tryptone	110	53	75
	yeast extract	87	181	76
Temperature	20	81	150	108
	25	110	177	175
	30	99	265	202
	35	56	145	154
	40	87	196	165

**Fig. 9:**
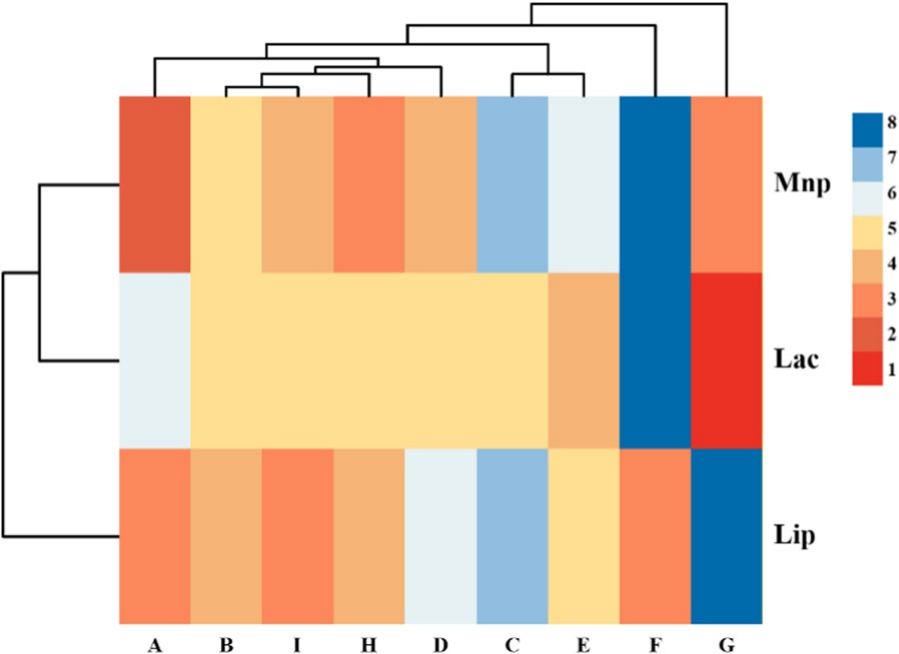
Effect of three factors on enzyme activity of QL-Z3. Medium Number: **A** 2.0 g/L lignin, pH = 11, NaNO_3_; **B** 2.0 g/L lignin, pH = 9.5, (NH_4_)_2_SO_4_; **C** 2.0 g/L lignin, pH = 8, NH_4_NO_3_; **D** 2.5 g/L lignin, pH = 11, (NH_4_)_2_SO_4_; **E** 2.5 g/L lignin, pH = 8, NaNO_3_; **F** 2.5 g/L lignin, pH = 9.5, NH_4_NO_3_; **G** 3.0 g/L lignin, pH = 8, (NH_4_)_2_SO_4_; **H** 3.0 g/L lignin, pH = 11, NH_4_NO_3_; **I** 3.0 g/L lignin, pH = 9.5, NaNO_3_. Each experimental group was conducted in triplicate

Based on the above results, the top three significant factors for supporting enzyme activity were lignin concentration, pH, nitrogen source. Next, we performed an L9 (3^3^), three-factor, three-level orthogonal experiment to test the effects of different combinations of these conditions. The optimal results are shown in Fig. 9, the orthogonal experimental listed from Additional file 1: Table S9-1 to Additional file 1: Table S9-6. The result validated that the optimal conditions of LiP production were 3 g/L alkaline lignin, pH = 8, nitrogen source was (NH_4_)_2_SO_4_, enzyme activity increase 3.57 time; Under the condition of 3 g/L alkaline lignin, pH = 8 and nitrogen source NH_4_NO_3_, the MnP activity of strain QL-Z3 was 839.5 U/L and Lac activity was 219.0 U/L, which were 3.18 and 2.84 times of those before optimization, respectively (Table 4).

**Table 4:** Comparison of the initial medium and optimized medium in enzyme activity

Enzyme	Initial medium (U/L)	Optimized medium (U/L)	Increase (%)
LiP	104.1	367.5	253
Lac	77.0	219.0	184
MnP	263.8	839.5	218

Compared with other strains, LiP, MnP and Lac have the advantages of short fermentation time, multiple and higher enzyme activities (Additional file 1: Table S10). The LiP, Lac and MnP activities in the crude enzyme solution of QL-Z3 fermentation were optimized to improve their enzyme activities, which applied for the production of biopharmaceuticals and industrial enzymes, such as laccase has great application potential as biocatalyst for papermaking, in environmental remediation, drug detection and biosensors in the clothing industry [56]; Peroxidase is widely used in wastewater treatment technology in paper production and clothing production [41]; These enzymes were discovered that can transform lignin-derived aromatic compounds have important theoretical and practical significance in alleviating environmental pollution caused by lignin, sustainable agricultural development and diversification of earth resources utilization.

## Conclusions

In this study, we reported lignin degrader *Erwinia billingiae* QL-Z3, of which degradation rate was 25.24% at 1.5 g/L lignin. Disruption of potential ligninolytic genes significantly reduced the lignin-degrading activity of QL-Z3 by 47–69%. The potential metabolic pathways were deduced by FTIR and LC–MS analysis according varies degradation products. Enzyme activities of LiP, MnP and Lac were 367.50 U/L, 839.50 U/L and 219.00 U/L by orthogonal optimization. In conclusion, our study provides new insights for the biological valorization of lignin into high-value bioproducts.

### Supplementary Information

**Additional file 1:** **Figure S1.** Morphological characterisation of *Erwinia billingiae* QL-Z3 by scanning electron microscopy (SEM). (scale bar = 3 µm). **Figure S2.** Effects of substrate concentration, nitrogen source, and pH on lignin degradation of *Erwinia billingiae* QL-Z3. **a** Lignin concentration; **b** pH; **c** Nitrogen source. Average values of three replicates are shown with the standard errors of the mean shown as error bars. Each experimental group was conducted in triplicate. **Figure S3.** Circular representation of the genome (chromosome and plasmid) of *Erwinia bilingiae* QL-Z3. **a** Chromosome; **b** Plasmid. **Figure S4.** Classification of eggNOG annotations of the *Erwinia bilingiae* QL-Z3 genome. **Figure S5.** KEGG pathway of the *Erwinia bilingiae* QL-Z3 genome. **Figure S6.** CAZy function classification of the *Erwinia bilingiae* QL-Z3 genome. **Figure S7.** Gene knockout PCR validation of the eight genes predicted to participate in lignin degradation. M1&M2&M3, 2 kb marker; 1,Negative control: △EDYP_48_QL-Z3; 2, △EDYP_48; 3, Positive control: △EDYP_48_S17-1; 4, Negative control: △ELAC_205_QL-Z3; 5, △ELAC_205; 6, Positive control: △ELAC_205_S17-1; 7, Negative control:△EDIO_858_QL-Z3; 8, △EDIO_858; 9, Positive control: △EDIO_858_S17-1; 10, Negative control: △EOXI_996_QL-Z3; 11, △EOXI_996; 12, Positive control:△EOXI_996_S17-1; 13, Negative control:△ESOD_1236_QL-Z3; 14, △ESOD_1236; 15, Positive control:△ESOD_1236_S17-1; 16, Negative control: △EMON_3330_QL-Z3; 17, △EMON_3330; 18, Positive control: △EMON_3330_S17-1; 19, Negative control: EMCAT_3587_QL-Z3; 20, EMCAT_3587; 21, Positive control: EMCAT_3587_S17-1; 22, Negative control: ECAT_3467_QL-Z3; 23, ECAT_3467; 24, Positive control: EMCAT_3467_S17-1. **Figure S8.** Complementation PCR validation of the eight genes predicted to participate in lignin degradation. Note: M, 2 kb Marker; 1, ΔEDYP_48 (EDYP_48); 2, △ELAC_205 (ELAC_205); 3, △EDIO_858 (EDIO_858); 4, △EOXI_996 (EOXI_996); 5, △ESOD_1236 (ESOD_1236); 6, △EMON_3330 (EMON_3330); 7, △ECAT_3467 (ECAT_3467); 8, △EMCAT_3587 (EMCAT_3587). **Figure S9.** Western Blot analysis of the six purified proteins predicted to participate in lignin degradation. M, Marker; 1, The precipitation of liquid; 2, Supernatant; 3–4, Outflow liquid; 5–11, Target protein. **a** EDYP_48; **b** ELAC_205; **c** EDIO_858; **d** ESOD_1236; **e** EMON_3330; **f** EMCAT_3587. **Figure S10.** Optimization results of enzyme activity conditions pH (**a**) Temperature (**b**) of EDYP_48 and ELAC_205. Blue: EDYP_48, red: ELAC_205. Average values of three replicates are shown with the standard errors of the mean shown as error bars. Each experimental group was conducted in triplicate. **Table S1-1.** L9 (3^3^) Test design of Degradation rate optimization. **Table S1-2.** Orthogonal model analysis of variance of Degradation rate optimization. **Table S2.** Comparison of degradability of lignin degrading microorganisms. **Table S3.** General features of *Erwinia bilingiae* QL-Z3 genome. **Table S4.** Primers used for gene knockout. **Table S5.** Primers used for gene complementation. **Table S6.** Gene real-time expression levels at 6 h and 72 h under lignin and starvation were shown. **Table S7.** Specific activities of heterologously expressed and purified ligninolytic enzymes. **Table S8.** The main degradation products of Alkali lignin obtained in different enzymes combinations. **Table S9-1.** L_9_ (3^3^) Test design of LiP. **Table S9-2.** Orthogonal model analysis of variance of LiP. **Table S9-3.** L_9_ (3^3^) Test design of Lac. **Table S9-4.** Orthogonal model analysis of variance of Lac. **Table S9-5.** L9 (3^3^) Test design of MnP. **Table S9-6.** Orthogonal model analysis of variance of MnP. **Table S10.** Comparison of three enzyme activities (LiP, MnP and Lac) of lignin-degrading microorganism.

## Data Availability

Data will be made available on request.
