# Supplementary material for: Lignin bioconversion based on genome mining for ligninolytic genes in Erwinia billingiae QL-Z3
Source: Biotechnol Biofuels Bioprod. 2024 Feb 15;17:25. doi: 10.1186/s13068-024-02470-z (PMC10870720; doi:10.1186/s13068-024-02470-z)
Supplement: Supplementary file 1 — Additional file 1: Figure S1. Morphological characterisation of Erwinia billingiae QL-Z3 by scanning electron microscopy (SEM). (scale bar = 3 µm). Figure S2. Effects of substrate concentration, nitrogen source, and pH on lignin degradation of Erwinia billingiae QL-Z3. a Lignin concentration; b pH; c Nitrogen source. Average values of three replicates are shown with the standard errors of the mean shown as error bars. Each experimental group was conducted in triplicate. Figure S3. Circular representation of the genome (chromosome and plasmid) of Erwinia bilingiae QL-Z3. a Chromosome; b Plasmid. Figure S4. Classification of eggNOG annotations of the Erwinia bilingiae QL-Z3 genome. Figure S5. KEGG pathway of the Erwinia bilingiae QL-Z3 genome. Figure S6. CAZy function classification of the Erwinia bilingiae QL-Z3 genome. Figure S7. Gene knockout PCR validation of the eight genes predicted to participate in lignin degradation. M1&M2&M3, 2 kb marker; 1,Negative control: △EDYP_48_QL-Z3; 2, △EDYP_48; 3, Positive control: △EDYP_48_S17-1; 4, Negative control: △ELAC_205_QL-Z3; 5, △ELAC_205; 6, Positive control: △ELAC_205_S17-1; 7, Negative control:△EDIO_858_QL-Z3; 8, △EDIO_858; 9, Positive control: △EDIO_858_S17-1; 10, Negative control: △EOXI_996_QL-Z3; 11, △EOXI_996; 12, Positive control:△EOXI_996_S17-1; 13, Negative control:△ESOD_1236_QL-Z3; 14, △ESOD_1236; 15, Positive control:△ESOD_1236_S17-1; 16, Negative control: △EMON_3330_QL-Z3; 17, △EMON_3330; 18, Positive control: △EMON_3330_S17-1; 19, Negative control: EMCAT_3587_QL-Z3; 20, EMCAT_3587; 21, Positive control: EMCAT_3587_S17-1; 22, Negative control: ECAT_3467_QL-Z3; 23, ECAT_3467; 24, Positive control: EMCAT_3467_S17-1. Figure S8. Complementation PCR validation of the eight genes predicted to participate in lignin degradation. Note: M, 2 kb Marker; 1, ΔEDYP_48 (EDYP_48); 2, △ELAC_205 (ELAC_205); 3, △EDIO_858 (EDIO_858); 4, △EOXI_996 (EOXI_996); 5, △ESOD_1236 (ESOD_1236); 6, △EMON_3330 (EMON_3330); 7, △ECAT_3467 (ECAT [file 13068_2024_2470_MOESM1_ESM.docx]

**Additional file 1**

**Lignin bioconversion based on genome mining for ligninolytic genes in *Erwinia*** ***billingiae* QL-Z3**

Shuting Zhao^1^, Dongtao Deng^1^, Tianzheng Wan^2^, Jie Feng^1^, Lei Deng^1^, Qianyi Tian^1^, Jiayu Wang^1^, Umm E Aiman^1^, Balym Mukhaddi^1^, Xiaofeng Hu^3^, Shaolin Chen^1^, Ling Qiu^4^, Lili Huang^5*^, Yahong Wei^1*^

^1^State Key Laboratory of Crop Stress Biology for Arid Areas, College of Life Sciences, Biomass Energy Center for Arid and Semi-Arid Lands, Northwest A&F University, Yangling, Shaanxi,712100, P. R. China.

^2^Vrije University Amsterdam, De Boelelaan 1105, 1081 HV Amsterdam, Netherlands.

^3^Shanghai Personal Biotechnology Co., Ltd, Shanghai 20030, P. R. China.

^4^College of Mechanical and Electronic Engineering, the West Scientific Observing and Experimental Station of Rural Renewable Energy Exploitation and Utilization of the Ministry of Agriculture, Northwest A&F University, Yangling, Shaanxi, 712100, P. R. China.

^5^State Key Laboratory of Crop Stress Biology for Arid Areas, College of Plant Protection, Northwest A&F University, Yangling, Shaanxi, 712100, P. R. China.


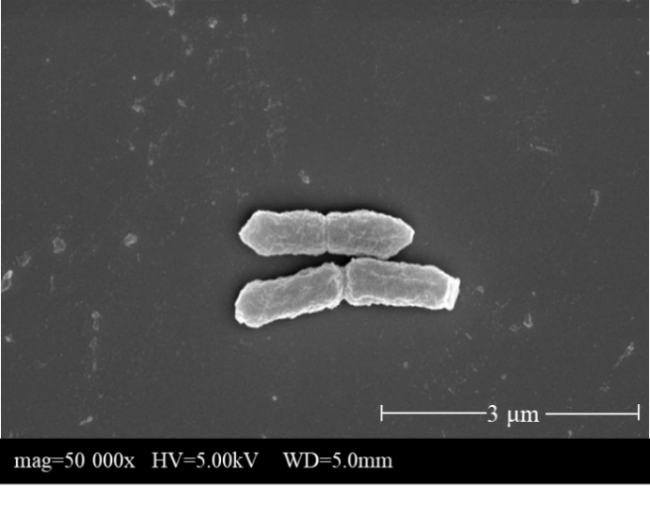


Figure S1. Morphological characterisation of *Erwinia billingiae* QL-Z3 by scanning electron microscopy (SEM). (scale bar = 3 µm).


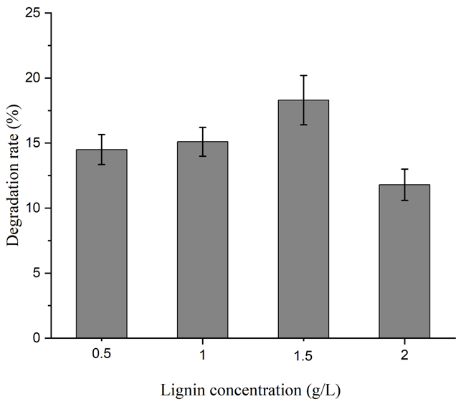

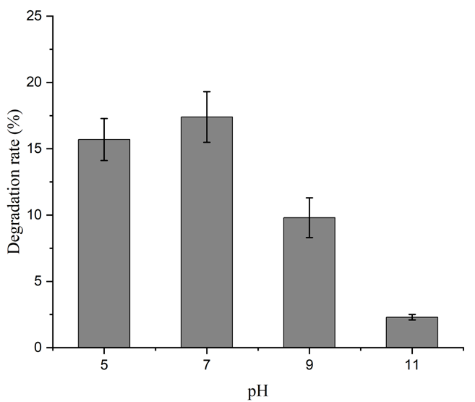


(a) (b)


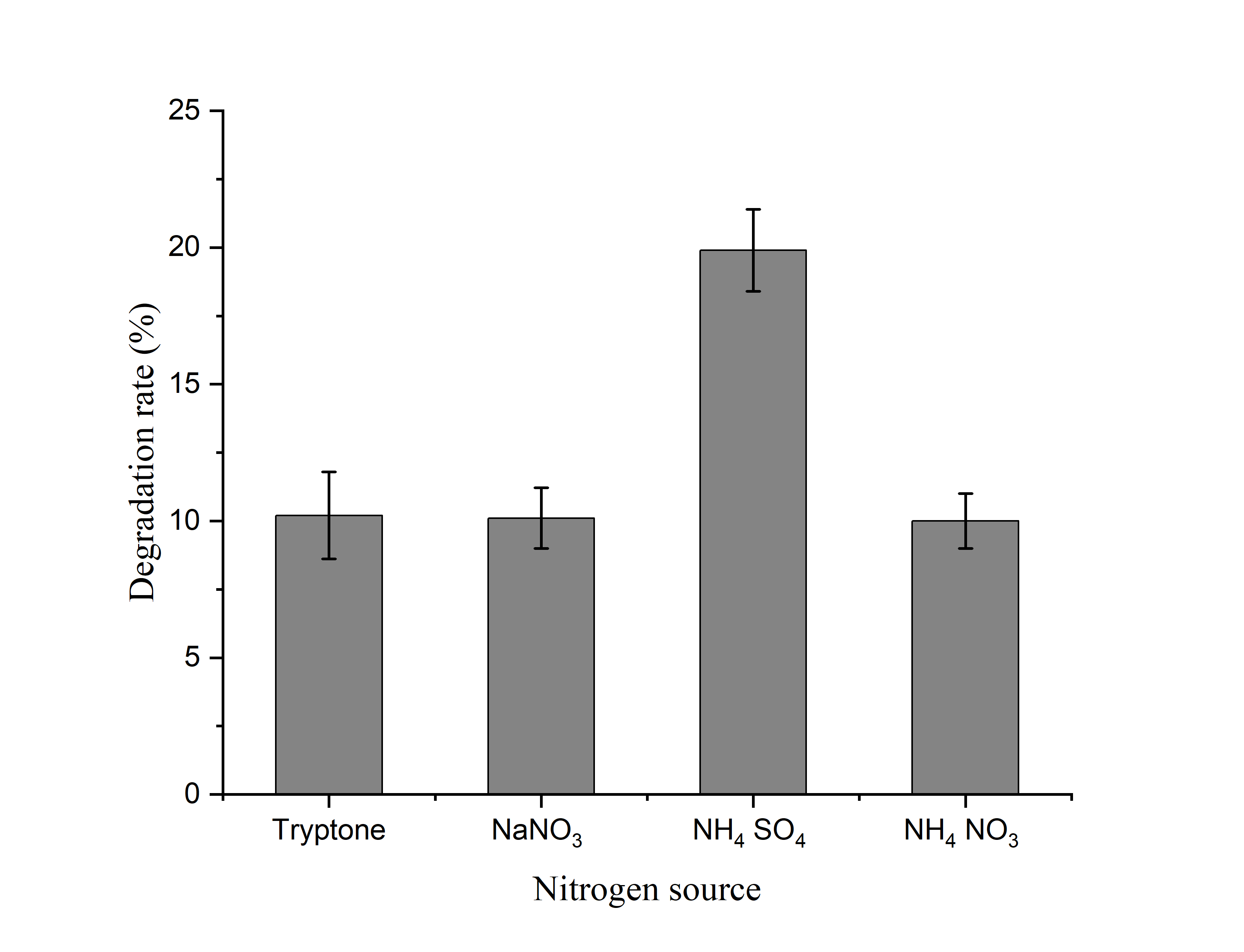


(c)

Figure S2. Effects of substrate concentration, nitrogen source, and pH on lignin degradation of *Erwinia billingiae* QL-Z3.

(a) Lignin concentration; (b) pH; (c) Nitrogen source. Average values of three replicates are shown with the standard errors of the mean shown as error bars. Each experimental group was conducted in triplicate.

(a)

(b)

Figure S3. Circular representation of the genome (chromosome and plasmid) of *Erwinia* *bilingiae* QL-Z3.

(a) Chromosome; (b) Plasmid.

Figure S4. Classification of eggNOG annotations of the *Erwinia bilingiae* QL-Z3 genome.

Figure S5. KEGG pathway of the *Erwinia* *bilingiae* QL-Z3 genome.

Figure S6. CAZy function classification of the *Erwinia* *bilingiae* QL-Z3 genome.


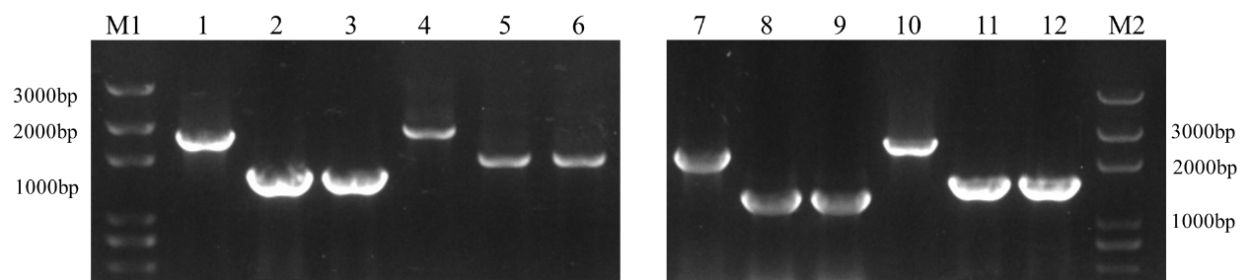


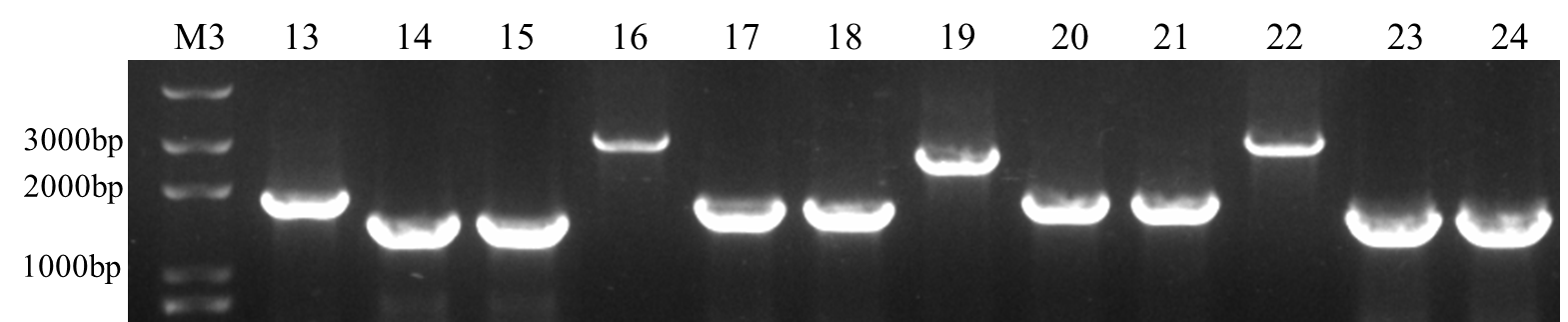


Figure S7. Gene knockout PCR validation of the eight genes predicted to participate in lignin degradation.

Note: M1＆M2＆M3, 2 kb marker; 1,Negative control: △EDYP_48_QL-Z3; 2, △EDYP_48; 3, Positive control: △EDYP_48_S17-1; 4, Negative control: △ELAC_205_QL-Z3; 5, △ELAC_205; 6, Positive control: △ELAC_205_S17-1; 7, Negative control:△EDIO_858_QL-Z3; 8, △EDIO_858; 9, Positive control: △EDIO_858_S17-1; 10, Negative control: △EOXI_996_QL-Z3; 11, △EOXI_996; 12, Positive control:△EOXI_996_S17-1; 13, Negative control:△ESOD_1236_QL-Z3; 14, △ESOD_1236; 15, Positive control:△ESOD_1236_S17-1; 16, Negative control: △EMON_3330_QL-Z3; 17, △EMON_3330; 18, Positive control: △EMON_3330_S17-1; 19, Negative control: EMCAT_3587_QL-Z3; 20, EMCAT_3587; 21, Positive control: EMCAT_3587_S17-1; 22, Negative control: ECAT_3467_QL-Z3; 23, ECAT_3467; 24, Positive control: EMCAT_3467_S17-1.


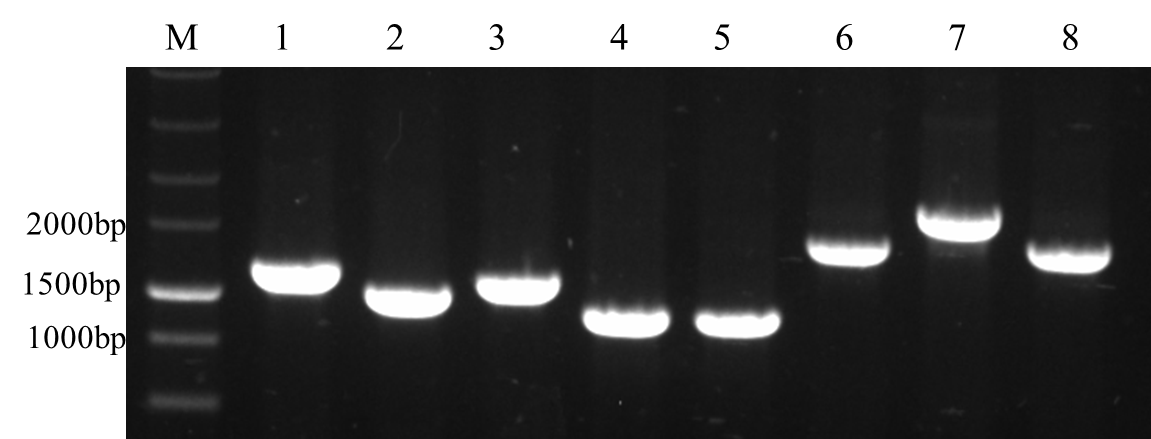


Figure S8. Complementation PCR validation of the eight genes predicted to participate in lignin degradation

Note: M, 2 kb Marker; 1, ΔEDYP_48 (EDYP_48); 2, △ELAC_205 (ELAC_205); 3, △EDIO_858 (EDIO_858); 4, △EOXI_996 (EOXI_996); 5, △ESOD_1236 (ESOD_1236); 6, △EMON_3330 (EMON_3330); 7, △ECAT_3467 (ECAT_3467); 8, △EMCAT_3587 (EMCAT_3587).


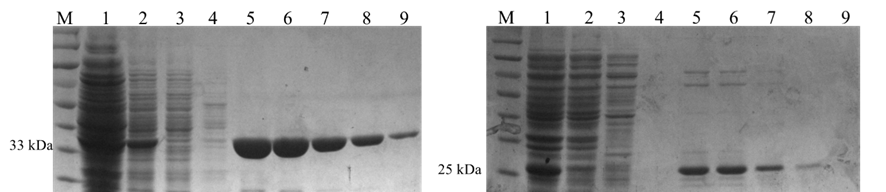


（a） （b）


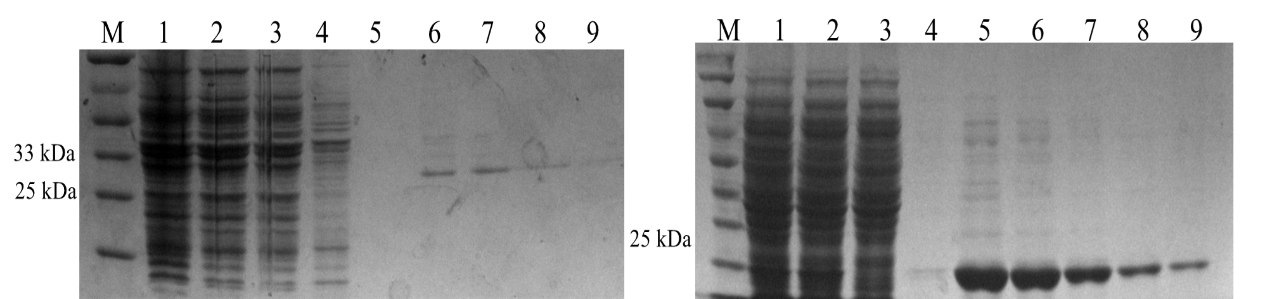


（c） （d）


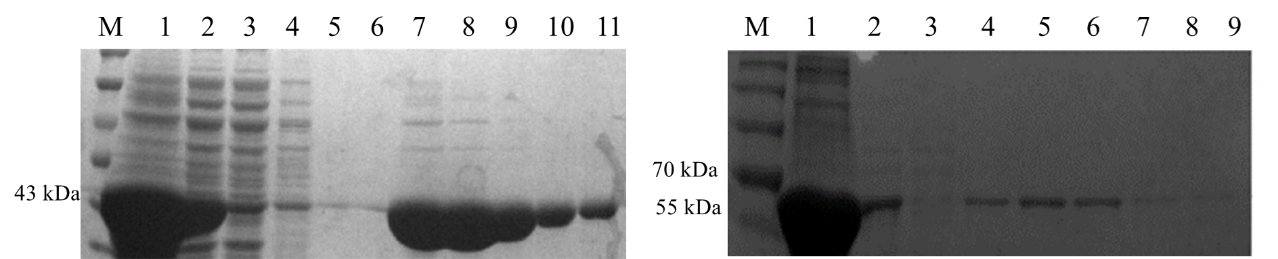


（e） （f）

Figure S9. Western Blot analysis of the six purified proteins predicted to participate in lignin degradation

Note: M, Marker; 1, The precipitation of liquid; 2, Supernatant; 3-4, Outflow liquid; 5-11, Target protein.

(a) EDYP_48; (b) ELAC_205; (c) EDIO_858; (d) ESOD_1236; (e) EMON_3330; (f) EMCAT_3587.


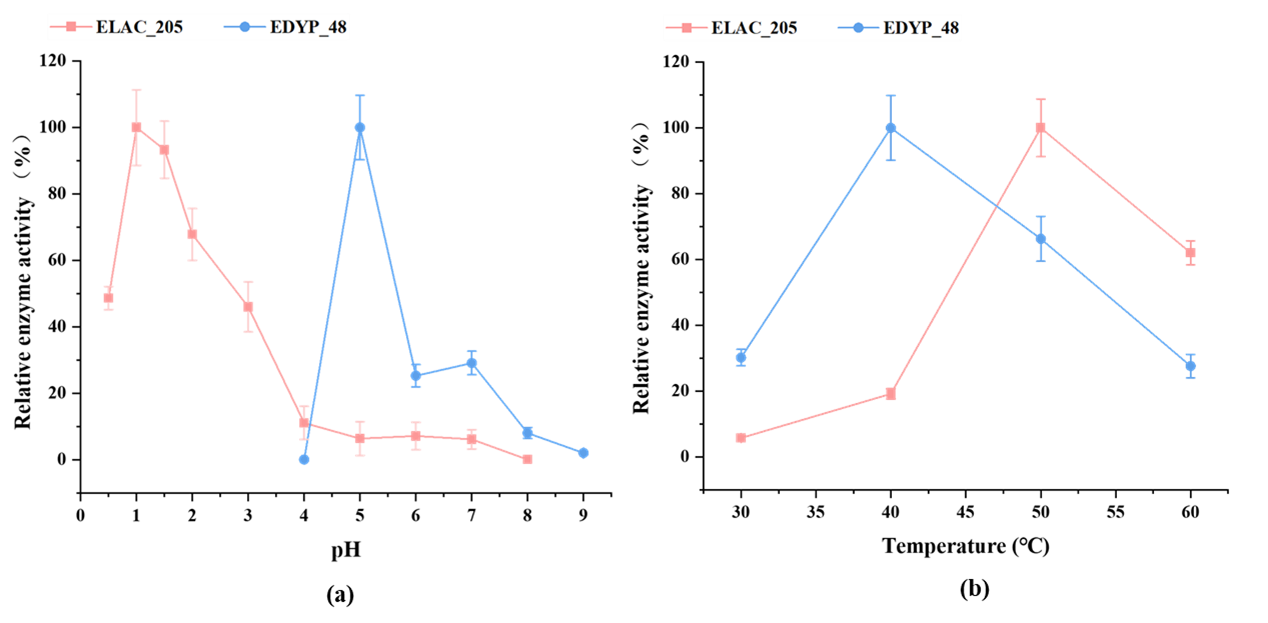


Figure S10. Optimization results of enzyme activity conditions pH (a) Temperature (b) of EDYP_48 and ELAC_205

Blue: EDYP_48, red: ELAC_205. Average values of three replicates are shown with the standard errors of the mean shown as error bars. Each experimental group was conducted in triplicate.

Table S1-1. L9 (3^3^) Test design of Degradation rate optimization

| Test number | Initial pH (A) | Nitrogen Source (B) | Lignin content(g·L^-1^) (C) | Degradation rate (%) |
| --- | --- | --- | --- | --- |
| 1 | 1(5) | 2((NH_4_)_2_SO_4_)（NH4)2SO4）（NH4)2SO4）（NaNO_3_） | 2(1.5) | 25.24 |
| 2 | 1(5) | 3(Tryptone) | 3(2) | 6.70 |
| 3 | 1(5) | 1(NaNO_3_) | 1(1) | 12.60 |
| 4 | 2(7) | 2((NH_4_)_2_SO_4_) | 1(1) | 11.40 |
| 5 | 2(7) | 1(NaNO_3_) | 3(2) | 4.59 |
| 6 | 2(7) | 3(Tryptone) | 2(1.5) | 19.64 |
| 7 | 3(9) | 1(NaNO_3_) | 2(1.5) | 10.63 |
| 8 | 3(9) | 3(Tryptone) | 1(1) | 3.03 |
| 9 | 3(9) | 2((NH_4_)_2_SO_4_) | 3(2) | 8.50 |
| K_1_ | 44.54 | 27.82 | 27.03 |  |
| K_2_ | 35.63 | 45.14 | 55.51 |  |
| K_3_ | 22.16 | 29.37 | 19.79 |  |
| k_1_ | 14.85 | 9.27 | 9.01 |  |
| k_2_ | 11.88 | 15.05 | 18.50 |  |
| k_3_ | 7.39 | 9.79 | 6.60 |  |
| Range R | 7.46 | 5.78 | 11.90 |  |
| Optimal Level | A_1_ | B_2_ | C_2_ |  |
| Primary and secondary | C>A>B | | |  |
| optimal combination | A_1_B_2_C_2_ | | |  |

Note: K_1_, K_2_ and K_3_ are the sum of degradation rates at 1, 2 and 3 levels corresponding to a single factor, k_1_, k_2_ and k_3_ are the corresponding average values respectively, and range R is the difference between the maximum K value and the minimum K value.

Table S1-2. Orthogonal model analysis of variance of Degradation rate optimization

| Project | Sum of squares of deviations | Freedom | Mean square | F value | P value | Significance |
| --- | --- | --- | --- | --- | --- | --- |
| Calibration model | 329.643^a^ | 6 | 54.940 | 21.026 | 0.033 | * |
| Intercept | 986.588 | 1 | 986.588 | 536.938 | 0.002 | ** |
| A | 137.144 | 2 | 68.572 | 37.320 | 0.026 | * |
| B | 101.276 | 2 | 50.638 | 27.559 | 0.035 | * |
| C | 91.222 | 2 | 45.611 | 24.823 | 0.039 | * |
| Error | 3.675 | 2 | 1.837 |  |  |  |
| Total | 1319.906 | 9 |  |  |  |  |
| Correction | 313.318 | 8 |  |  |  |  |
| Coefficient | R^2^=0.989(Adjusted for R^2^=0.956） | | | | | |

Note: **, "P < 0.01" means extremely significant difference; *, "P < 0.05" means significant difference

Table S2. Comparison of degradability of lignin degrading microorganisms

| Microorganism | Fermentation conditions | Type of lignin | Lignin concentration (g/L) | Degradation rate (%) | References |
| --- | --- | --- | --- | --- | --- |
| *Erwinia billingiae* QL-Z3 | 30℃/3d | Alkali lignin | 1.5 | 25.24 | This study |
| *Bacillus flexus* RMWW II | 35℃/9d | Alkali lignin | 0.1 | 97.10 | [S1] |
| *Brevibacillus thermoruber* | 55°C/5d | Alkali lignin | 0.385 | 81.90 | [S2] |
| *Acetoanaerobium* sp. WJDL-Y2 | 30℃/12d | Kraft lignin | 1.2 | 24.90 | [S3] |
| *Pseudomonas* sp. Q18 | 37℃/14d | Raw lignocellulosc | 3 | 35.00 | [S4] |
| *Ochrobactrum oryzae* BMP03 | 30℃/14d | Powdered rice straw | 3 | 53.74 | [S5] |
| *Odontotermes obesus* IIPTG13 | 35℃/14d | Guaiacylglycerol-b-guaiacyl | 0.1 | 61.33 | [S6] |
| *Comamonas* sp. B-9 | 30℃/7d | Kraft lignin | 3 | 32 | [S7] |
| *Rhodococcus pyridinivorans*  CCZU-B16 | 30℃/3d | Alkali lignin | 4 | 30.2 | [S8] |
| *Novosphingobium* sp. B-7 | 30℃/7d | Kraft lignin | 3 | 34.7 | [S9] |
| *Lentinus edodes* | 30℃/10d | Lignin of raw black liquor | 0.92 | 65 | [S10] |
| *Aspergillus* sp. F-3 | 30℃/8d | Alkali lignin | 2 | 65 | [S11] |
| *Streptomyces* spp. F-6 | 30℃/12d | Alkali lignin | 2 | 50 | [S12] |
| *Phanerochaete sordida* YK-624 | 30℃/10d | Extractive-free beech wood | 0.5 | 32.7 | [S13] |
| *Phanerochaete chrysosporium* | 35℃/36d | Lignin of maize straw | 0.22 | 64.30 | [S14] |

Table S3. General features of *Erwinia* *bilingiae* QL-Z3 genome

| Features | Chromosome | Plasmid |
| --- | --- | --- |
| Genome size (bp) | 4286943 | 108924 |
| G + C content (%) | 56.25% | 54.67% |
| rRNAs | 22 | 0 |
| tRNAs | 78 | 0 |
| Total predicted CDSs | 4556 | 166 |
| Genes with predicted functions (NR) | 4428 | 140 |

Table S4. Primers used for gene knockout

| Primer name | 5’-3’sequence | Length (bp) |
| --- | --- | --- |
| 48F1 | GCTCTAGAGATTGGCGTCGGGCTGTATCAG | 812 |
| 48R1 | AGCCACACCTACGCGATAAGCCCCGCAC |  |
| 48F2 | TATCGCGTAGGTGTGGCTCTCCTGCTATT | 816 |
| 48R2 | CCGCTCGAGGCTGTACTGGCTGGCTTTCA |  |
| 205F1 | GCTCTAGACCGGACTCAGCACGTTCG | 862 |
| 205R1 | TGGACTGGTACCTAGCGCCATAAGACGGTC |  |
| 205F2 | GCGCTAGGTACCAGTCCATCTGATCCTTGAATTC | 843 |
| 205R2 | CCGCTCGAGGTCATCAACAAACCACGCGG |  |
| 858F1 | GCTCTAGAATGACAGCAGAACAGCAGTATC | 736 |
| 858R1 | AGTACACTCGGGCTGTGAATTAGTCAGCG |  |
| 858F2 | TCACAGCCCGAGTGTACTCCGTGCCAC | 640 |
| 858R2 | CCGCTCGAGTGCTGACCATTGCCAGTTC |  |
| 996F1 | GCTCTAGAGAGGAAGGTGGCGATCTTGT | 988 |
| 996R1 | ATTATTGCACATTGATGATGCGTTAGGCATA |  |
| 996F2 | ATCAATGTGCAATAAT GCGCCTGG | 601 |
| 996R2 | CCGCTCGAGCATCCAGCACCACATAACG |  |
| 1236F1 | GCTCTAGACATTCCGGTATCGGCACTGCTG | 647 |
| 1236R1 | GCGGGGATTTAATGGTGGGCGGCGCTG |  |
| 1236F2 | ACCATTAAATCCCCGCCACGTTAATTCG | 513 |
| 1236R2 | CCGCTCGAGGAAATGAATACGCCTGAGGGTG |  |
| 3330F1 | GCTCTAGAGCGACATTTAGCCGGTAATAG | 783 |
| 3330R1 | CGCTCATGACTATCCGTTTCCTATATAACGATATCTG |  |
| 3330F2 | GAAACGGATAGTCATGAGCGATCGGCATATTG | 860 |
| 3330R2 | CCGCTCGAGACAGGCGTTGACCGTTGAG |  |
| 3587F1 | GCTCTAGAAGTCGTGGACATGGAAGATATTAGC | 971 |
| 3587R1 | AGTAAGCAAGCATTCAGTGCAAGGGCAG |  |
| 3587F2 | TGAATGCTTGCTTACTCCGGATAACCAGTAT | 951 |
| 3587R2 | CCGCTCGAGAGGGCACTGTAAAGACATCAAC |  |
| 3467F1 | GCTCTAGAACGTTGTCTTGCAGCAG | 1423 |
| 3467R1 | GCTGCGTTTCCATTGTTGTCTCTCCGACTTA |  |
| 3467F2 | GACAACAATGGAAACGCAGCTAATCCTCTTA | 1460 |
| 3467R2 | CCGCTCGAGGCGCCGTAACAATTTCCATGA |  |

Table S5. Primers used for gene complementation

| Primers | 5’-3’sequence | Length  (bp) |
| --- | --- | --- |
| 48 F | GCTCTAGAATGTCCCAGTCCCAGAGCG | 812 |
| 48 R | CGGAATTCTTACAGGGCGGCTAACTGCTG |  |
| 205 F | AACTGCAGATGAGCCTGATCACGCCTGAC | 862 |
| 205 R | ACGCGTCGACTTATATCAGCCAGATCAAACTTGCCAG |  |
| 858 F | GCTCTAGAATGAACGAACGTCTGACGATTACC | 736 |
| 858 R | CGGAATTCTCAGCGTTCAGCGCGGTAAAAAG |  |
| 996 F | GCTCTAGAATGTCGGTCTGTGTCGTTGG | 988 |
| 996 R | CGGAATTCTTATTGCGGCTCCTTTTTCGC |  |
| 1236 F | AACTGCAGATGGAGATGATGAACATGAGTTATTCACTG | 647 |
| 1236 R | ACGCGTCGACTTATTTTGCTGCTGAGAAAAGCGCTG |  |
| 3330 F | GCTCTAGAATGAGCGACGCAGCGC | 783 |
| 3330 R | CGCTCATGACTATCCGTTTCCTATATAACGATATCTG |  |
| 3587 F | CGGAATTCTCAGTTTCCCTGTTTGCCCG | 951 |
| 3587 R | CGGAATTCCTATTTTTTCGCCGGCTTGCC |  |
| 3467 F | GTACCCTCGAGGGATCCGAATTCATGAGCAATGATCCACGTAAAC | 1470 |
| 3467 R | CTAGACTGCAGGTCGACAAGCTTTTAGCTGCGTTTCGC |  |
| pKT F | TCCTGGTGTCCCTGTTGATACCG | 306 |
| pKT R | CGCCAGGGTTTTCCCAGTCA |  |

Table S6. Gene real-time expression levels at 6h and 72h under lignin and starvation were shown

| gene ID | Real-time quantification | | | |
| --- | --- | --- | --- | --- |
|  | Lignin (6h) | Starvation (6h) | Lignin (72h) | Starvation (72h) |
| Chr_468 | 0.86±0.05 | 1.07±0.13 | 0.92±0.01 | 0.42±0.06 |
| Chr_2803 | 0.73±0.10 | 0.84±0.03 | 0.85±0.05 | 0.18±0.007 |
| Chr_3132 | 0.98±0.12 | 0.66±0.05 | 0.23±0.002 | 0.74±0.07 |

Collected Ct values of lignin, glucose and starvation group of different genes at 6h and 72h, then analyzed and calculated the relative expression levels of corresponding target genes in different samples. Finally, the expression level of genes in the glucose group was set as the control value 1, the relative expression levels of the lignin and starvation group were calculated respectively when glucose was used as the control. Each experimental group was conducted in triplicate.

Table S7. Specific activities of heterologously expressed and purified ligninolytic enzymes

| Name | Encoding protein | Specific enzyme activity  (U/mg) (±SD) |
| --- | --- | --- |
| EDYP_48 | Dyp-type peroxidase | 2.73 ± 0.32 |
| ELAC_205 | Laccase | 0.11 ± 0.01 |
| EDIO_858 | Dioxygenase | 25.36 ± 3.29 |
| ESOD_1236 | Superoxide Dismutase | 31.35 ± 2.47 |
| EMON_3330 | Monooxygenase | 45.6 ± 4.88 |
| EMCAT_3587 | Manganese Catalase | 33.78 ± 3.68 |

Table S8. The main degradation products of Alkali lignin obtained in different enzymes combinations

| NO. | Structural formula | Name | Enzymatic hydrolysis combination | | | | | | |
| --- | --- | --- | --- | --- | --- | --- | --- | --- | --- |
|  |  |  | L | D | S | LD | LS | DS | LDS |
| 1 | 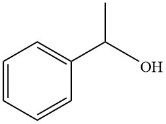 | 1-Phenylethanol | **+++** | **++** | **+** | **+++** | **++** | **++** | **+++** |
| 2 | 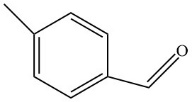 | 4-Methylbenzaldehyde | **++** |  | **++** | **++** | **+++** | **++** | **++** |
| 3 | 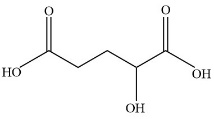 | 2-Hydroxyglutarate | **++** | **+** | **++** | **++** | **++** | **++** | **++** |
| 4 | 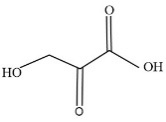 | Hydroxypyruvic acid | **++** |  | **++** |  | **++** | **++** | **+** |
| 5 | 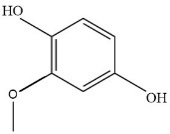 | 2-Methoxyhydro-quinone | **++** | **+** | **+** | **++** | **++** | **+** | **++** |
| 6 | 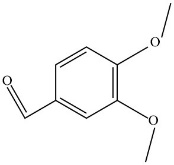 | 3,4-Dimethoxybenzaldehyde | **+** |  | **++** | **++** | **+++** | **++** | **++** |
| 7 | 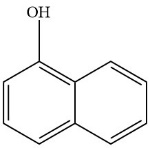 | 1-Naphthol | **+** | **+** | **+** |  |  |  |  |
| 8 | 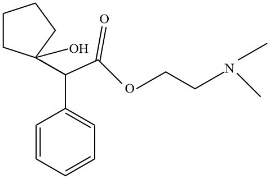 | Cyclopentolate | **+** | **+** |  | **+** | **+** |  | **+** |
| 9 | 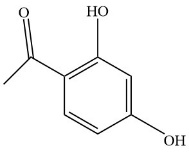 | 2,4-Dihydroxyaceto-phenone | **+** |  | **++** |  | **++** | **++** | **++** |
| 10 | 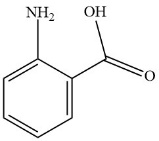 | 2-Aminobenzoic acid | **+** |  |  |  |  |  |  |
| 11 | 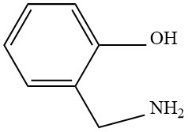 | 2-(Aminomethyl) phenol | **+** |  |  | **+** |  |  |  |
| 12 | 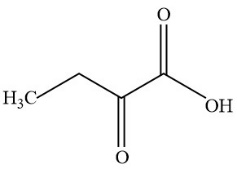 | 2-Ketobutyric acid | **-** |  | **+** | **-** |  | **+** | **-** |
| 13 | 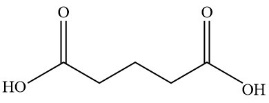 | Glutaric acid | **-** | **-** | **-** | **-** | **-** | **-** | **-** |
| 14 | 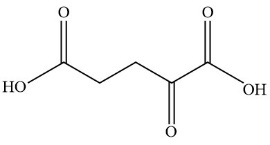 | Oxoglutaric acid | **-** |  |  | **-** | **-** | **-** | **-** |
| 15 | 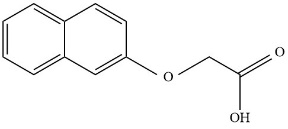 | 2-Naphthalenyl-oxyacetic acid | **-** | **+** |  | **-** | **+** |  |  |
| 16 | 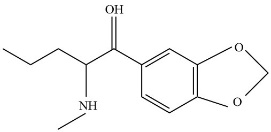 | Pentylone | **-** | **-** | **-** | **-** | **-** | **-** | **-** |
| 17 | 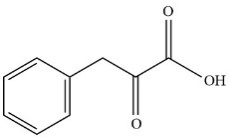 | Phenylpyruvic acid | **-** | **-** | **-** | **-** | **-** | **-** | **-** |
| 18 | 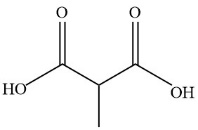 | Methylmalonic acid |  |  |  |  |  | **-** |  |
| 19 | 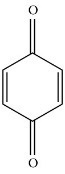 | Quinone |  |  |  | **+** | **+** |  | **+** |
| 20 | 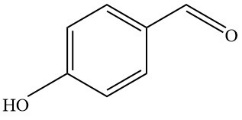 | 4-Hydroxybenzal-dehyde |  | **+** |  | **+** |  |  |  |
| 21 | 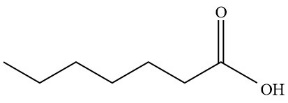 | Heptanoic acid |  |  |  |  | **-** |  |  |
| 22 | 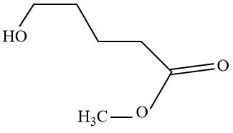 | Monomethyl glutaric acid |  | **+** | **+** |  |  | **+** | **+** |
| 23 | 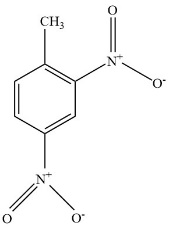 | 2,4-Dinitrotoluene |  |  |  |  |  |  | **+** |
| 24 | 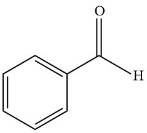 | Benzaldehyde |  | **+** |  |  |  |  |  |
| 25 | 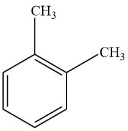 | O-Xylene |  |  | **++** |  |  |  |  |
| 26 | 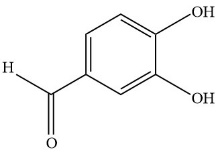 | 3,4-Dihydroxybenzaldehye |  |  | **-** |  |  |  |  |
| 27 | 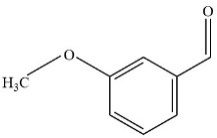 | 3-Methoxybenzaldehyde |  | **+** |  |  | **+** |  |  |
| 28 | 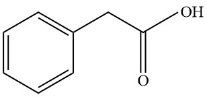 | Phenylacetic acid |  |  | **-** |  | **-** | **-** |  |
| 29 | 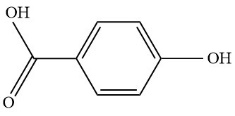 | 4-Hydroxybenzoic acid |  |  |  |  | **+** |  |  |
| 30 | 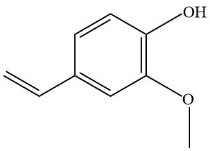 | 2-Methoxy-4-vinylphenol |  |  |  |  | **-** |  |  |
| 31 | 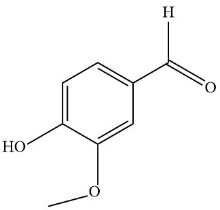 | 4-Hydroxy-3-methoxy-benzaldehyde |  | **+** |  |  |  |  |  |
| 32 | 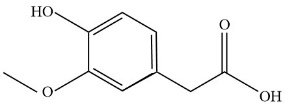 | Homovanillic acid |  | **-** | **-** |  | **-** | **-** |  |
| 33 | 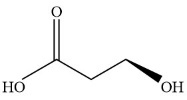 | (R)-3-Hydroxybutyric acid | **+** |  |  |  | **+** |  |  |
| 34 | 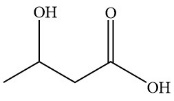 | 3-Hydroxybutyric acid | **+** |  |  | **+** |  |  | **+** |
| 35 | 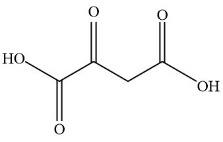 | Oxalacetic acid | **-** | **-** | **-** | **-** | **-** | **-** | **-** |
| 36 | 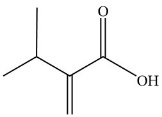 | Alpha-Ketoisovaleric acid | **-** |  | **+** | **-** |  | **+** | **-** |
| 37 | 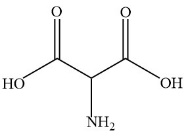 | Aminomalonic acid |  |  | **+** | **+** | **+** |  |  |
| 38 | 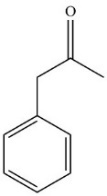 | Phenylacetaldehyde | **+** | **+** | **+** | **+** | **+** | **+** | **+** |
| 39 | 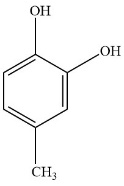 | 4-Methylcatechol |  | **-** |  |  |  |  |  |
| 40 | 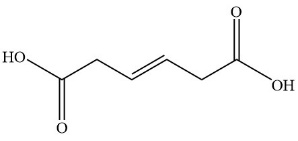 | 3-Hexenedioic acid | **+** | **+** |  |  | **+** | **+** |  |
| 41 | 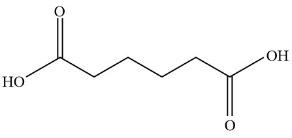 | Adipic acid | **+** |  |  |  |  | **+** |  |
| 42 | 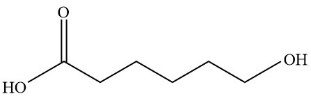 | 6-Hydroxyhexanoic acid | **++** | **+** | **+** | **+** | **+** | **+** | **+** |
| 43 | 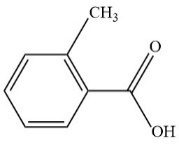 | O-Toluate |  | **+** |  |  |  | **+** | **-** |
| 44 | 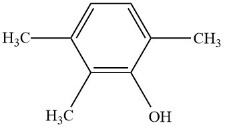 | 2,3,6-Trimethylphenol |  |  | **-** |  | **-** |  |  |
| 45 | 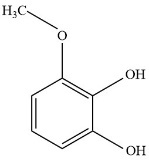 | 3-methoxycatechol | **+** |  | **+** | **+** | **+** | **+** | **+** |
| 46 | 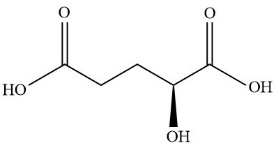 | L-2-Hydroxyglutaric acid | **-** |  |  |  |  |  |  |
| 47 | 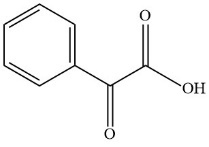 | Phenylglyoxylic acid | **+** |  | **+** | **+** |  |  | **+** |
| 48 | 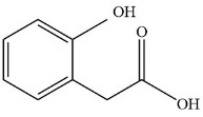 | Ortho-Hydroxyphenylacetic acid |  |  |  | **+** | **+** |  | **+** |
| 49 | 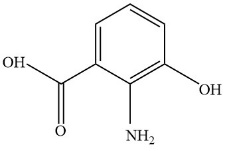 | 3-Hydroxyanthranilic acid | **+** | **+** | **-** | **+** |  |  | **+** |
| 50 | 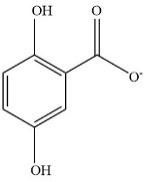 | 2,5-dihydroxybenzoate |  |  |  | **+** | **-** |  |  |
| 51 | 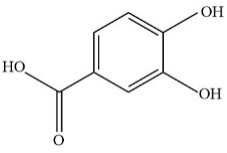 | Protocatechuic acid | **+++** | **+++** | **+++** | **+++** | **+++** | **+++** | **+++** |
| 52 | 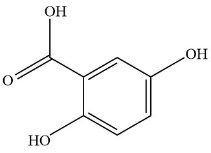 | 2,5-Dihydroxybenzoic acid Gentisic acid |  |  |  | **-** |  |  |  |
| 53 | 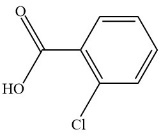 | 2-Chlorobenzoate acid | **++** | **-** | **+++** | **++** | **+++** | **+++** | **+++** |
| 54 | 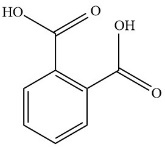 | Phthalic acid | **++** | **++** |  | **++** | **++** | **++** |  |
| 55 | 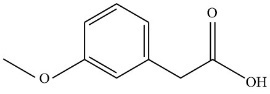 | 3-Methoxyphenylacetic acid | **+** |  |  |  |  |  | **+** |
| 56 | 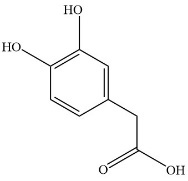 | 3,4-Dihydroxy-benzeneacetic acid | **-** | **-** | **-** | **-** | **-** | **-** | **-** |
| 57 | 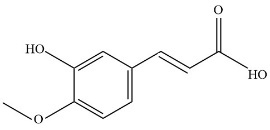 | Isoferulic acid |  | **+** | **-** | **+** |  |  |  |
| 58 | 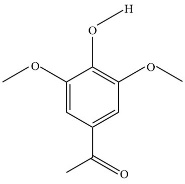 | Acetosyringone |  |  |  |  |  |  | **-** |
| 59 | 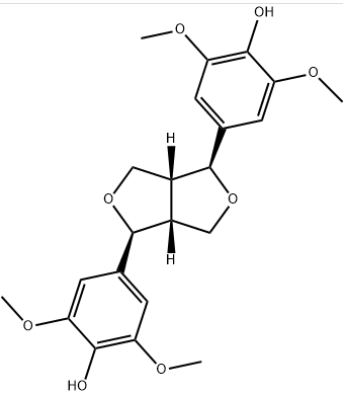 | (+)-Syringaresinol |  |  | **+** |  |  |  |  |
| 60 | 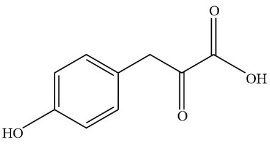 | 4-Hydroxyphenylpyruvic acid | **+** | **+** |  | **+** |  | **+** |  |
| 61 | 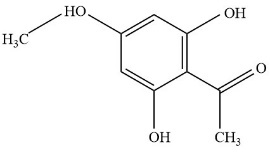 | 2',6'-Dihydroxy-4'-methoxyacetophenone |  | **+** |  |  |  |  |  |
| 62 | 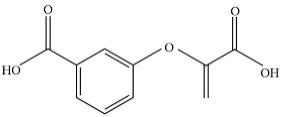 | 3-[(1-Carboxyvinyl) oxy] benzoic acid |  |  |  |  |  | **+** |  |
| 63 | 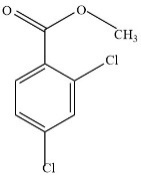 | 2,4-dichlorobenzoate | **+** |  |  | **+** |  |  |  |
| 64 | 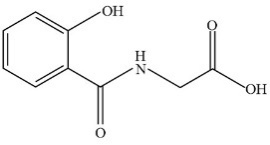 | Salicyluric acid | **+** |  |  | **+** | **+** |  | **+** |
| 65 | 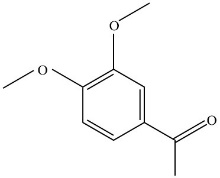 | 3,4-Dimethoxyacetophenone | **+** |  |  | **+** |  |  |  |
| 66 | 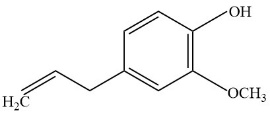 | Eugenol |  |  | **-** |  | **-** |  |  |
| 67 | 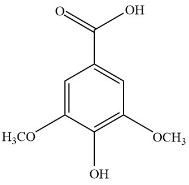 | Syringic acid | **+** | **+** |  | **+** | **+** | **+** | **+** |
| 68 | 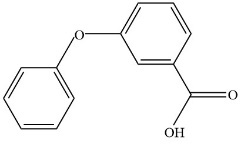 | 3-phenoxybenzoic acid | **+** | **+** |  | **+** | **+** | **+** |  |
| 69 | 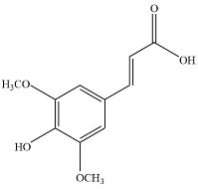 | Sinapic acid | **+** | **+** |  | **+** |  |  |  |
| 70 | 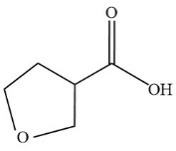 | 3-Furoic acid |  |  | **+** | **+** | **+** | **+** |  |
| 71 | 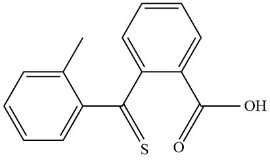 | 2-[2-(methylthio)benzoyl]  benzoic acid | **+** | **+** |  | **+** |  |  | **+** |
| 72 | 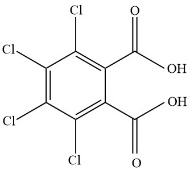 | Tetrachlorophthalic acid | **-** |  | **-** | **-** | **-** | **-** | **-** |
| 73 | 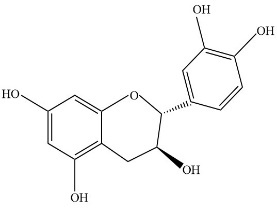 | Catechin | **+++** | **+++** | **+++** | **+++** | **+++** | **+++** | **+++** |
| 74 | 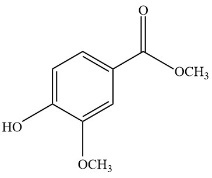 | Methyl vanillate | **-** |  | **-** |  | **-** | **-** |  |
| 75 | 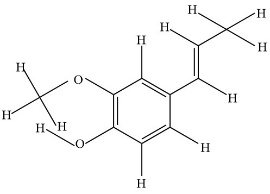 | 4-Propenyl-2-methoxyphenol | **-** | **+** | **-** | **+** | **-** | **-** | **-** |
| 76 | 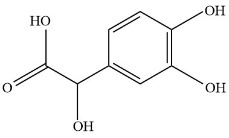 | 3,4-Dihydroxymandelic acid | **-** | **-** |  | **-** | **+** | **-** | **-** |
| 77 | 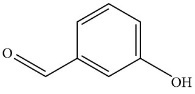 | 3-Hydroxybenzal-dehyde |  |  |  | **+** | **+** |  |  |
| 78 | 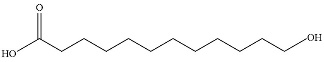 | 12-Hydroxydodecanoic acid | **+** | **+** | **+** | **+** | **+** | **+** | **+** |
| 79 | 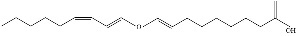 | 8-Nonenoic acid | **++** | **++** | **+++** | **++** | **+++** | **+++** | **++** |
| 80 | 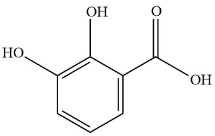 | 2,3-Dihydroxybenzoic acid |  |  |  | **+** |  | **+** |  |
| 81 | 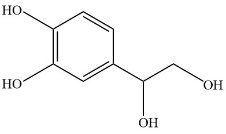 | 3,4-Dihydroxyphenyl-glycol |  | **+** |  | **+** |  | **+** | **+** |
| 82 | 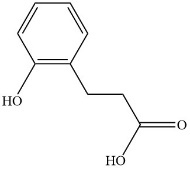 | 3-(2-Hydroxyphenyl) propanoic acid |  |  |  | **-** | **-** |  | **-** |
| 83 | 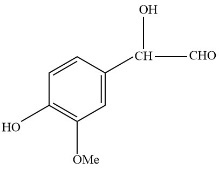 | 3-Methoxy-4-hydroxyphenylglycolaldehyde |  |  | **-** | **-** | **-** |  | **-** |
| 84 | 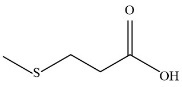 | 3-Methylthiopropionic acid | **-** | **-** | **-** |  | **-** | **-** | **-** |
| 85 | 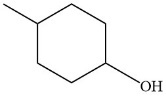 | 4-Methylbenzyl alcohol | **++** | **++** | **+++** | **++** | **+++** | **+++** | **++** |
| 86 | 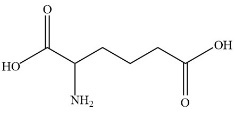 | Aminoadipic acid |  |  |  |  |  | **+** |  |
| 87 | 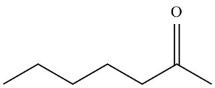 | Caproic acid | **+++** | **+** | **+++** | **+++** | **+++** | **+++** | **+++** |
| 88 | 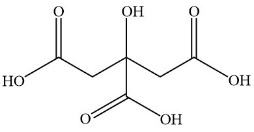 | Citric acid | **-** | **-** | **-** | **-** | **-** | **-** | **-** |

“+” indicates that this degradation product is up-regulated than control, one “+” means fold change of treatment group vs control was 1~3, “++” means 3-10, “+++”means Greater than 10;

“-” indicates that this degradation product is down-regulated than control;

Blank space indicates no product generation.

Table S9-1. L_9_ (3^3^) Test design of LiP

| Test number | Lignin content (A)(g·L^-1^) | Nitrogen source(B) | Initial pH (C) | LiP enzyme U/L |
| --- | --- | --- | --- | --- |
| 1 | 1(2.0) | 2(NaNO_3_) | 3(11.0) | 164.50 |
| 2 | 1(2.0) | 3((NH_4_)_2_SO_4_) | 2(9.5) | 218.40 |
| 3 | 1(2.0) | 1(NH_4_NO_3_) | 1(8.0) | 303.00 |
| 4 | 2(2.5) | 3((NH_4_)_2_SO_4_) | 3(11.0) | 276.50 |
| 5 | 2(2.5) | 2(NaNO_3_) | 1(8.0) | 264.50 |
| 6 | 2(2.5) | 1(NH_4_NO_3_) | 2(9.5) | 167.50 |
| 7 | 3(3.0) | 3((NH_4_)_2_SO_4_) | 1(8.0) | 371.50 |
| 8 | 3(3.0) | 1(NH_4_NO_3_) | 3(11.0) | 210.50 |
| 9 | 3(3.0) | 2(NaNO_3_) | 2(9.5) | 194.50 |
| K_1_ | 685.90 | 681.00 | 939.00 |  |
| K_2_ | 708.50 | 623.50 | 580.40 |  |
| K_3_ | 776.50 | 866.40 | 651.50 |  |
| k_1_ | 238.63 | 303.67 | 273.00 |  |
| k_2_ | 302.83 | 174.50 | 226.80 |  |
| k_3_ | 225.50 | 288.80 | 267.17 |  |
| Range R | 64.20 | 129.17 | 55.83 |  |
| Optimal Level | A_3_ | B_3_ | C_1_ |  |
| Primary and secondary |  | B>A>C |  | |
| Optimal  combination | A_3_B_3_C_1_ | | |  |

Note: K_1_, K_2_ and K_3_ are the sum of degradation rates at 1, 2 and 3 levels corresponding to a single factor, k_1_, k_2_ and k_3_ are the corresponding average values respectively, and range R is the difference between the maximum K value and the minimum K value.

Table S9-2. Orthogonal model analysis of variance of LiP

| Project | Sum of squares of deviations | Freedom | Mean square | F value | P value | Significance |
| --- | --- | --- | --- | --- | --- | --- |
| Calibration model | 54692.040^a^ | 6 | 9115.340 | 26.573 | 0.037 | * |
| Intercept | 514041.201 | 1 | 514041.201 | 1498.512 | 0.001 | ** |
| A | 24321.236 | 2 | 12160.618 | 0.509 | 0.027 | * |
| B | 25026.202 | 2 | 12513.101 | 0.842 | 0.027 | * |
| C | 5344.602 | 2 | 2672.301 | 1.304 | 0.114 |  |
| Error | 686.069 | 2 | 343.034 |  |  |  |
| Total | 569419.310 | 9 |  |  |  |  |
| Correction | 55378.109 | 8 |  |  |  |  |
| Coefficient | R^2^=0.988(Adjusted for R^2^=0.950） | | | | | |

Note: **, "P < 0.01" means extremely significant difference; *, "P < 0.05" means significant difference.

Table S9-3. L_9_ (3^3^) Test design of Lac

| Test number | Lignin content (A)(g·L^-1^) | Nitrogen source (B) | Initial pH (C) | Lac enzyme  U/L |
| --- | --- | --- | --- | --- |
| 1 | 1(2.0) | 2(NaNO_3_) | 3(11.0) | 190.50 |
| 2 | 1(2.0) | 3((NH_4_)_2_SO_4_) | 2(9.5) | 149.00 |
| 3 | 1(2.0) | 1(NH_4_NO_3_) | 1(8.0) | 159.00 |
| 4 | 2(2.5) | 3((NH_4_)_2_SO_4_) | 3(11.0) | 167.00 |
| 5 | 2(2.5) | 2(NaNO_3_) | 1(8.0) | 135.44 |
| 6 | 2(2.5) | 1(NH_4_NO_3_) | 2(9.5) | 219.00 |
| 7 | 3(3.0) | 3((NH_4_)_2_SO_4_) | 1(8.0) | 59.17 |
| 8 | 3(3.0) | 1(NH_4_NO_3_) | 3(11.0) | 158.67 |
| 9 | 3(3.0) | 2(NaNO_3_) | 2(9.5) | 151.33 |
| K_1_ | 498.50 | 536.67 | 353.61 |  |
| K_2_ | 521.44 | 477.27 | 519.33 |  |
| K_3_ | 369.17 | 375.17 | 516.17 |  |
| k_1_ | 166.17 | 178.89 | 117.87 |  |
| k_2_ | 173.81 | 159.09 | 173.11 |  |
| k_3_ | 123.06 | 125.06 | 172.06 |  |
| Range R | 50.76 | 53.83 | 55.24 |  |
| Optimal Level | A_2_ | B_1_ | C_2_ |  |
| Primary and secondary | C>B>A | | |  |
| Optimal  combination | A_2_B_1_C_2_ | | |  |

Note: K_1_, K_2_ and K_3_ are the sum of degradation rates at 1, 2 and 3 levels corresponding to a single factor, k_1_, k_2_ and k_3_ are the corresponding average values respectively, and range R is the difference between the maximum K value and the minimum K value.

Table S9-4. Orthogonal model analysis of variance of Lac

| Project | Sum of squares of deviations | Freedom | Mean square | F value | P value | Significance |
| --- | --- | --- | --- | --- | --- | --- |
| Calibration model | 14930.280^a^ | 6 | 2488.380 | 23.583 | 0.041 | * |
| Intercept | 214402.955 | 1 | 214402.955 | 2031.916 | 0.000 | ** |
| A | 4493.183 | 2 | 2246.591 | 21.291 | 0.045 | * |
| B | 4448.336 | 2 | 2224.168 | 21.079 | 0.045 | * |
| C | 5988.762 | 2 | 2994.381 | 28.378 | 0.034 | * |
| Error | 211.035 | 2 | 105.518 |  |  |  |
| Total | 229544.270 | 9 |  |  |  |  |
| Correction | 15141.316 | 8 |  |  |  |  |
| Coefficient | R^2^=0.986(Adjusted for R^2^=0.944） | | | | | |

Note: **, "P < 0.01" means extremely significant difference; *, "P < 0.05" means significant difference.

Table S9-5. L9 (3^3^) Test design of MnP

| Test Number | Lignin content (A)(g·L^-1^) | Nitrogen source (B) | Initial pH (C) | MnP enzyme  U/L |
| --- | --- | --- | --- | --- |
| 1 | 1(2.0) | 2(NaNO_3_) | 3(11.0) | 245.00 |
| 2 | 1(2.0) | 3((NH_4_)_2_SO_4_) | 2(9.5) | 470.83 |
| 3 | 1(2.0) | 1(NH_4_NO_3_) | 1(8.0) | 728.75 |
| 4 | 2(2.5) | 3((NH_4_)_2_SO_4_) | 3(11.0) | 396.67 |
| 5 | 2(2.5) | 2(NaNO_3_) | 1(8.0) | 650.50 |
| 6 | 2(2.5) | 1(NH_4_NO_3_) | 2(9.5) | 839.50 |
| 7 | 3(3.0) | 3((NH_4_)_2_SO_4_) | 1(8.0) | 338.75 |
| 8 | 3(3.0) | 1(NH_4_NO_3_) | 3(11.0) | 285.00 |
| 9 | 3(3.0) | 2(NaNO_3_) | 2(9.5) | 443.44 |
| K_1_ | 1444.58 | 1853.25 | 1718.00 |  |
| K_2_ | 1886.67 | 1338.94 | 1753.77 |  |
| K_3_ | 1067.19 | 1206.25 | 926.67 |  |
| k_1_ | 481.53 | 617.75 | 572.67 |  |
| k_2_ | 628.89 | 446.31 | 584.59 |  |
| k_3_ | 355.73 | 402.08 | 308.89 |  |
| Range R | 273.16 | 215.67 | 275.70 |  |
| Optimal Level | A_2_ | B_1_ | C_2_ |  |
| Primary and secondary | C>A>B | | |  |
| Optimal  combination | A_2_B_1_C_2_ | | |  |

Note: K_1_, K_2_ and K_3_ are the sum of degradation rates at 1, 2 and 3 levels corresponding to a single factor, k_1_, k_2_ and k_3_ are the corresponding average values respectively, and range R is the difference between the maximum K value and the minimum K value.

Table S9-6. Orthogonal model analysis of variance of MnP

| Project | Sum of squares of deviations | Freedom | Mean square | F value | P value | Significance |
| --- | --- | --- | --- | --- | --- | --- |
| Calibration model | 335746.859^a^ | 6 | 55957.810 | 21.026 | 0.046 | * |
| Intercept | 2149586.048 | 1 | 2149586.048 | 807.701 | 0.001 | ** |
| A | 112157.139 | 2 | 56078.569 | 21.071 | 0.045 | * |
| B | 77858.935 | 2 | 38929.467 | 14.628 | 0.064 |  |
| C | 145730.786 | 2 | 72865.393 | 27.379 | 0.035 | * |
| Error | 5322.729 | 2 | 2661.364 |  |  |  |
| Total | 2490655.636 | 9 |  |  |  |  |
| Correction | 341069.588 | 8 |  |  |  |  |
| Coefficient | R^2^=0.984(Adjusted for R^2^=0.938） | | | | | |

Note: **, "P < 0.01" means extremely significant difference; *, "P < 0.05" means significant difference.

Table S10. Comparison of three enzyme activities (LiP、MnP and Lac) of lignin-degrading microorganism

| Microorganism | Fermentation conditions | | LiP (U/L) | Lac (U/L) | MnP (U/L) | References |
| --- | --- | --- | --- | --- | --- | --- |
| *Erwinia billingiae* QL-Z3 | 30℃/3d | | 367.5 | 219.0 | 839.5 | This study |
| *Brevibacillus thermoruber* | 55 °C/3-5d | | 984.5 | 67.3 | 90.7 | [S2] |
| *Comamonas* sp. B-9 | 30℃/4-6d | | / | 1250.0 | 2903.2 | [S7] |
| *Novosphingobium* sp. B-7 | 30℃/7d | | / | 1275 | 3229.8 | [S9] |
| *Aspergillus* sp. F-3 | 30℃/7d | / | | 3.5 | 28.2 | [S11] |
| *Arthrobacter* sp. C2 | 15℃/7d | | 29.5 | / | 52.4 | [S15] |
| *Pantoea* sp. F3h | 15°C/7d | | 21.3 | / | 30.5 | [S15] |
| *Pseudomonas* sp. M6 | 15℃/7d | | 24.8 | / | 35.4 | [S15] |
| *Cupriavidus basilensis* B-8 | 30℃/3-4d | | / | 815.6 | 1685.3 | [S16] |
| *Streptomyces thermocarboxydus* DF3-3 | 30℃/4-7d | | 480.3 | 1265.6 | 1821.7 | [36] |

**References of supplementary materials**

[S1] Kumar A, Priyadarshinee R, Singha S, Sengupta B, Roy A, Dasgupta D, Mandal T. Biodegradation of alkali lignin by *Bacillus flexus* RMWW II: analyzing performance for abatement of rice mill wastewater. Water Science and Technology. 2019; 80(9): 1623-1632.

[S2] Niu J, Li X, Qi X, Ren Y. Pathway analysis of the biodegradation of lignin by *Brevibacillus thermoruber*. Bioresource Technology. 2021; 341: 125875.

[S3] Duan J, Huo X, Du WJ, Liang JD, Wang DQ, Yang SC. Biodegradation of kraft lignin by a newly isolated anaerobic bacterial strain. *Acetoanaerobium* sp. WJDL-Y2. Lett Appl Microbiol. 2016; 62(1): 55-62.

[S4] Yang C, Yue F, Cui Y, Xu Y, Shan Y, Liu B, Zhou Y, Lü X. Biodegradation of lignin by *Pseudomonas* sp. Q18 and the characterization of a novel bacterial DyP-type peroxidase. J Ind Microbiol Biotechnol. 2018; 45(10): 913-927.

[S5] Tsegaye B, Balomajumder C, Roy P. Biodelignification and hydrolysis of rice straw by novel bacteria isolated from wood feeding termite. 3 Biotech. 2018; 8(10): 447.

[S6] Sunil KS, Manisha D, Deependra T, Vimee R, Dilip KA, Pankaj KK. Investigation of lignin biodegradation by *Trabulsiella* sp. isolated from termite gut, International Biodeterioration & Biodegradation. 2016; 112: 12-17.

[S7] Chen YH, Chai LY, Zhu YH, Yang ZH, Zheng Y, Zhang H. Biodegradation of kraft lignin by a bacterial strain *Comamonas* sp. B-9 isolated from eroded bamboo slips. J Appl Microbiol. 2012; 112(5):900-6.

[S8] Chong GG, Huang XJ, Di JH, Xu DZ, He YC, Pei YN, Tang YJ, Ma CL. Biodegradation of alkali lignin by a newly isolated *Rhodococcus pyridinivorans* CCZU-B16. Bioprocess Biosyst Eng. 2018; 41(4): 501-510.

[S9] Chen YH, Chai LY, Tang CJ, Yang ZH, Zheng Y, Shi Y, Zhang H. Kraft lignin biodegradation by *Novosphingobium* sp. B-7 and analysis of the degradation process, Bioresource Technology. 2012; 123: 682-685.

[S10] Wu J, Xiao YZ, Yu HQ. Degradation of lignin in pulp mill wastewaters by white-rot fungi on biofilm. Bioresour Technol. 2005; 96(12): 1357-63.

[S11] Yang YS, Zhou JT, Lu H, Yuan YL, Zhao LH. Isolation and characterization of a fungus *Aspergillus* sp. strain F-3 capable of degrading alkali lignin. Biodegradation. 2011; 22(5): 1017-27.

[S12] Yang YS, Zhou JT, Lu H, Yuan YL, Zhao LH. Isolation and characterization of *Streptomyces* spp. strains F-6 and F-7 capable of decomposing alkali lignin. Environ Technol. 2012; 33(22-24):2603-9.

[S13] Wang J, Suzuki T, Mori T, Yin R, Dohra H, Kawagishi H, Hirai H. Transcriptomics analysis reveals the high biodegradation efficiency of white-rot fungus *Phanerochaete sordida* YK-624 on native lignin. J Biosci Bioeng. 2021; 132(3): 253-257.

[S14] Chen Y, Wang Y, Xu Z, Liu Y, Duan H. Enhanced humification of maize straw and canola residue during composting by inoculating *Phanerochaete chrysosporium* in the cooling period. Bioresour Technol. 2019; 293: 122075.

[S15] Cheng Y, Huang M, Shen X, Jiang C. Enhanced cornstalk decomposition by a psychrotrophic bacterial consortium comprising cellulose, hemicellulose, and lignin degraders with biochar as a carrier for carbonneutrality. Bioresour Technol. 2022; 344: 126259.

[S16] Shi Y, Chai L, Tang C, Yang Z, Zhang H, Chen R, Chen Y, Zheng Y. Characterization and genomic analysis of kraft lignin biodegradation by the beta-proteobacterium *Cupriavidus basilensis* B-8. Biotechnol Biofuels. 2013; 6(1): 1.
